# APEX1, a transcriptional hub for endochondral ossification and fracture repair

**DOI:** 10.1038/s41413-025-00486-1

**Published:** 2026-01-16

**Authors:** José Valdés-Fernández, Miguel Echanove-González de Anleo, Juan Antonio Romero-Torrecilla, Tania López-Martínez, Purificación Ripalda-Cemboráin, María Erendira Calleja-Cervantes, Asier Ullate-Agote, Elena Iglesias, Belén Prados-Pinto, José Luis de la Pompa, Felipe Prósper, Emma Muiños-López, Froilán Granero-Moltó

**Affiliations:** 1 Cell Therapy Area, Clínica Universidad de Navarra, Pamplona, Spain; 2 Department of Orthopedic Surgery and Traumatology, Clínica Universidad de Navarra, Pamplona, Spain; 3 Hemato-Oncology Program, Centre for Applied Medical Research (CIMA), Universidad de Navarra, Pamplona, Spain; 4 Instituto de Investigación Sanitaria de Navarra (IdiSNA), Pamplona, Spain; 5 Biomedical Engineering Program, Centre for Applied Medical Research (CIMA), Universidad de Navarra, Pamplona, Spain; 6 Intercellular Signaling in Cardiovascular Development and Disease Laboratory, Centro Nacional de Investigaciones Cardiovasculares Carlos III (CNIC), Madrid, Spain; 7 Ciber Cardiovascular, Instituto de Salud Carlos III, Madrid, Spain; 8 Department of Hematology, Clínica Universidad de Navarra, Pamplona, Spain

**Keywords:** Bone, Pathogenesis

## Abstract

After injury, bone tissue initiates a reparative response to restore its structure and function. The failure to initiate or delay this response could result in fracture nonunion. The molecular mechanisms underlying the occurrence of fracture nonunion are not yet established. We propose that hypoxia-triggered signaling pathways, mediated by reactive oxygen species (ROS) homeostasis, control *Bmp2* expression and fracture healing initiation. The excessive ROS leads to oxidative stress and, ultimately, fracture nonunion. In this study, we silenced *Apex1*, the final ROS signaling transducer that mediates the activation of key transcription factors by their cysteines oxidoreduction, evaluating its role during endochondral ossification and fracture repair. Silencing *Apex1* in limb bud mesenchyme results in transient metaphyseal dysplasia derived from impaired chondrocyte differentiation. During bone regeneration, *Apex1* silencing induces a fracture nonunion phenotype, characterized by delayed fracture repair initiation, impaired periosteal response, and reduced chondrocyte and osteoblast differentiation. This compromised chondrocyte differentiation hampers callus vascularization and healing progression. Our findings highlight a critical mechanism where hypoxia-driven ROS signaling in mesenchymal progenitors through APEX1 is essential for fracture healing initiation.

## Introduction

Bone tissue presents truly regenerative capacity, as it is capable of repairing itself without scarring. This efficient system of tissue regeneration recapitulates different aspects of bone development and the two modalities of bone formation, intramembranous and endochondral ossification, are present during the healing process.^1^

A major complication of bone regeneration is fracture nonunion, which represents a major clinical challenge for orthopedic surgeons. Between 1% and 3% of the 178 million annual fractures registered globally result in fracture nonunion. Local factors such as fracture type, location, blood supply, or presence of infection are major determinants of fracture nonunion. In addition, systemic factors present in the patient at the time of fracture may increase the risk of fracture healing complications, delayed union, and nonunion,^2–5^ such as smoking, alcohol consumption, diabetes, chronic inflammation, anti-inflammatory medications, corticosteroids, or aging.

There are two primary types of aseptic fracture nonunion, atrophic and hypertrophic, each believed to originate from different mechanisms and requiring specific treatment approaches. In hypertrophic nonunions, a biological response is present, identified as a fracture callus, but the fractured bones lack sufficient stability, leading to incomplete healing. In consequence, the treatment requires changes in the fixation strategy to increase mechanical stability. Atrophic nonunions are more challenging, as the biological response is either absent or suboptimal, requiring the addition of bone grafts, growth factors or osteogenic cells to restore the biological reaction.^6,7^

At the molecular level, the causes of fracture nonunion are not yet elucidated. Fracture healing is a complex process that involves the overlapping and coordinated participation of various cell types, cellular processes, and signaling pathways through three sequential phases: inflammatory, reparative and remodeling.^1,8^ Disruption of any of these factors during the healing process could result in different levels of delayed union or fracture nonunion.

Genetic murine models have provided important clues to the molecular pathways and the cellular types implicated in both types of nonunion. BMP signaling, and specifically *Bmp2* expression by mesenchymal progenitors during the inflammatory phase, is recognized as the critical event in the initiation of fracture healing through periosteal activation.^9,10^ Silencing *Bmp2* in limb mesenchyme impairs fracture healing initiation, a phenotype that resembles an atrophic nonunion.^11,12^ Experimentally, inducing hypertrophic nonunion in mice is challenging, as rodents can heal fractures in the absence of mechanical stability. Nevertheless, delayed repair and hypertrophic-like phenotypes have been observed when specific matrix metalloproteases are silenced, hindering callus vascularization and fracture healing progression by compromising cartilage intermediate resorption.^13–16^

We and others have proposed that, at the molecular level, oxidative stress is a key factor in the development of fracture nonunion, and specifically atrophic nonunion.^17–19^ Immediately after trauma, vascular damage results in hematoma formation, producing localized hypoxia in the fracture line and surrounding tissues. At the cellular level, hypoxia triggers the formation of reactive oxygen species (ROS) by the mitochondria.^20,21^ We proposed that in mesenchymal progenitors, ROS-driven hypoxia signaling determines fracture healing initiation through control of *Bmp2* expression.^17^ This physiological mechanism of fracture healing initiation could be inhibited when ROS scavenging systems fail or are overcome, resulting in supraphysiological levels of ROS and oxidative stress, damaging the cell in different compartments, including lipids, proteins, and DNA.^22,23^

We found that the scavenging function carried out by the thioredoxin system was critical for *Bmp2/BMP2* expression and fracture healing initiation.^17^ The thioredoxin system is also one of the primary disulfide-reducing systems and a major mechanism of intracellular signaling. Under physiological conditions, ROS regulate different cellular processes, such as cell differentiation, cell proliferation, and the response to oxidative stress itself. ROS also modulate the activity of transcription factors such as AP-1 (FOS/JUN heterodimer), NF-κB, or HIF-1α, which are known to play critical roles in endochondral ossification and the fracture healing process.^24–28^ The regulation for these transcription factors requires the translocation of thioredoxin (TXN) to the nucleus and its interaction with APEX1, mediating the reduction of specific disulfide bonds, enabling DNA binding and the final activation of transcription factors.^29^ APEX1 is a bifunctional protein, initially identified by its apurinic/apyrimidinic endonuclease activity (APE-1) participating in the base excision repair (BER) pathway, and later recognized independently as REF-1 for its oxidoreductase activity.^30,31^ Together, these findings point to the critical interplay of hypoxia-induced ROS signaling and the TXN-APEX1 system in determining *Bmp2* expression during fracture healing initiation and preventing nonunion.

Here, we silenced *Apex1* in mice to specifically impair the transcriptional control initiated by ROS without affecting the scavenging function of TXN, and evaluated the role of this signaling pathway in endochondral ossification, fracture healing initiation and progression.

## Results

### Apex1 silencing in limb bud mesenchyme impacts bone development

To determine *Apex1* expression pattern in mouse appendicular skeleton, we used Apex1^tm1a^ KO first allele that functions as a reporter tagged insertion allele, where Apex1 promoter drives the transcription of bacterial β-galactosidase (Fig. S1a).^32^ Genetic tracking of endogenous *Apex1* expression revealed that, in young adult mice, *Apex1* is expressed in chondrocytes along the growth plate, suggesting that its silencing may disrupt bone tissue development and growth (Fig. S1b).

**Fig. 1:**
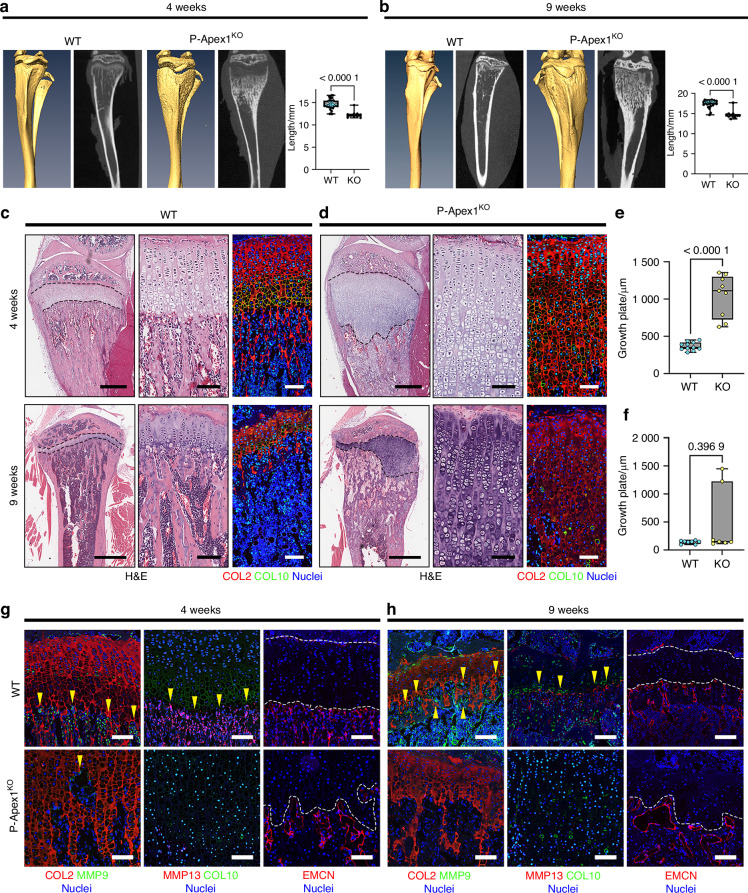
*Apex1* silencing in limb bud mesenchyme results in metaphyseal dysplasia. **a** Representative 3D reconstruction, radiographic plane and general morphometric parameters for 4-week-old mice tibiae (WT, *n* = 36; P-Apex1^KO^, *n* = 10). **b** Representative 3D reconstruction, radiographic plane and general morphometric parameters for 9-week-old mice tibiae (WT, *n* = 36; P-Apex1^KO^, *n* = 10). Data are represented as median and interquartile range; whiskers represent maximal and minimal values. *P* values were determined by two-tailed Student’s t test. **c** Representative H&E staining and collagen distribution of tibia samples for 4-week-old mice. Dotted black lines label the edges of the growth plate. Scale bar: left panels, 500 μm; central and right panels, 100 μm. **d** Histomorphometric determination of growth plate length in 4-week-old animals. (WT, *n* = 11; P-Apex1^KO^, *n* = 9). Data are represented as median and interquartile range; whiskers represent maximal and minimal values. *P* values were determined by two-tailed Student’s t test. **e** Representative H&E staining and collagen distribution for tibiae of 9-week-old mice. Scale bar, left panels,1 mm; central and right panels, 100 μm. **f** Histomorphometric determination of growth plate length in 9-week-old animals (WT, *n* = 8; P-Apex1^KO^, *n* = 7). Data are represented as median and interquartile range; whiskers represent maximal and minimal values. *P* values were determined by Mann-Whitney U test. **g**, **h** Immunological evaluation of chondrocyte differentiation, cartilage degradation, and vascularization markers in the growth plate of 4-week-old mice (**g**) and 9-week-old mice (**h**). Scale bar: 100 μm. COL2, type II collagen; COL10, type X collagen; EMCN, Endomucin. Dotted white lines mark the chondro-osseous junction

To investigate the role of *Apex1* during bone development, we examined the effects of *Apex1* silencing through endochondral ossification by using Prrx1-Cre mice, in which Cre recombinase is expressed in limb mesenchyme starting at E9.5.^33^ We crossed Apex1^C/C^ mice with Prrx1-Cre mice (Prrx1-Cre;Apex1^C/+^), the resulting Prrx1-Cre;Apex^C/C^ (P-Apex1^KO^) mice were born at the expected mendelian ratio, and no major morphological differences were appreciated when compared to wild-type (WT) littermates. However, after weaning (at 4 weeks old), radiological and morphometric analysis showed that animals exhibited an expanded growth plate in the long-bones, wider metaphysis, increased radiodensity below the expanded radiolucent growth plate, and shortening of the limbs, all signs of metaphyseal chondrodysplasia (Fig. 1a, Fig. S1c).^34^ Radiological analysis of the tibia in adult animals (9-weeks-old) showed that while the reduced bone length persisted (Fig. 1b), morphometric differences and radiolucent growth plate resolved in 8 out of 12 mice (Fig. 1b, Fig. S1d).

Histological and immunohistological analysis of the growth plate cartilage confirmed the radiological findings. At 4 weeks of age, WT animals displayed a well-defined growth plate with distinct resting, proliferative, prehypertrophic, and hypertrophic zones (Fig. S2a, b). Regarding cartilage components, WT animals presented abundant type II collagen deposition throughout the growth plate, while type X collagen was localized in the terminal region, clearly marking the hypertrophic zone (Fig. 1c). Conversely, while P-Apex1^KO^ mice presented similar resting and proliferative zones, the hypertrophic zone was significantly enlarged (Fig. S2a, b). Consequently, type II collagen was similarly present along the growth plate with no differences in the transcription level of *Col2a1* (Fig. S2c), whereas type X collagen deposition was inhibited, and a defined hypertrophic zone could not be delimited (Fig. 1d).

Histological analysis at 9 weeks revealed that all the animals presented a normal developmental shortening of the growth plate, although P-Apex1^KO^ mice still had columns of unresolved cartilage. In WT animals, the quantification of the areas stained for both type II and X collagen was reduced, with type X collagen marking the end of the shortened hypertrophic zone. In P-Apex1^KO^ mice, deposition of type X collagen remained reduced, even in areas where cartilage degradation was apparent (Fig. 1e, f).

Impaired cartilage resorption in P-Apex1^KO^ mice suggested a potential disruption in osteoclast/chondroclast activity or recruitment. However, quantification of the TRAP signal did not show significant differences between WT and P-Apex1^KO^ mice (Fig. S3), suggesting that impaired chondrocyte differentiation could be the driver of growth plate expansion and delayed resorption.

To determine if the metalloprotease-mediated cartilage degradation was affected by *Apex1* silencing, we performed double immunofluorescence for markers of cartilage maturation along with MMP-9 and MMP-13. Additionally, we visualized the vascularization of the tibia, directly dependent on cartilage resorption, by type H endothelium detection with Endomucin.^35^

At 4 weeks, double immunofluorescence for MMP-13 and type X collagen in WT mice revealed a strong accumulation of MMP-13 in the chondro-osseous junction, with delimitation of the hypertrophic zone. In P-Apex1^KO^ mice the signals for both markers of hypertrophy were reduced in the growth plate. Similarly, double immune fluorescence for type II collagen and MMP-9 in WT mice showed strong MMP-9 reactivity at the chondro-osseous junction of the growth plate. On the other hand, P-Apex1^KO^ mice exhibited a lack of MMP-9 signal along the elongated growth plate, with only a reduced presence of MMP-9 detected in its distal part, next to the trabecular region. Analysis of type H vessels disposition revealed that while WT animals presented a dense, organized network of type H vessels at the chondro-osseous junction, P-Apex1^KO^ mice displayed enlarged type H vessels outlining an irregular chondro-osseous junction (Fig. 1g).

At 9 weeks, in WT animals, there was an increase in MMP-13 and MMP-9 activity together with a dense network of type H vessels invading the bone marrow space and delineating the chondro-osseous junction. In P-Apex1^KO^ mice, the hypertrophic zone was not differentiated, MMP-13 and MMP-9 were barely detectable, and the type H vessels were enlarged and irregularly outlined the chondro-osseous junction (Fig. 1h).

In summary, silencing *Apex1* in the limb mesenchyme results in impaired chondrocyte differentiation impacting the normal endochondral ossification process and longitudinal bone growth.

### Apex1 silencing in limb mesenchyme delays fracture healing causing nonunion

**Fig. 2:**
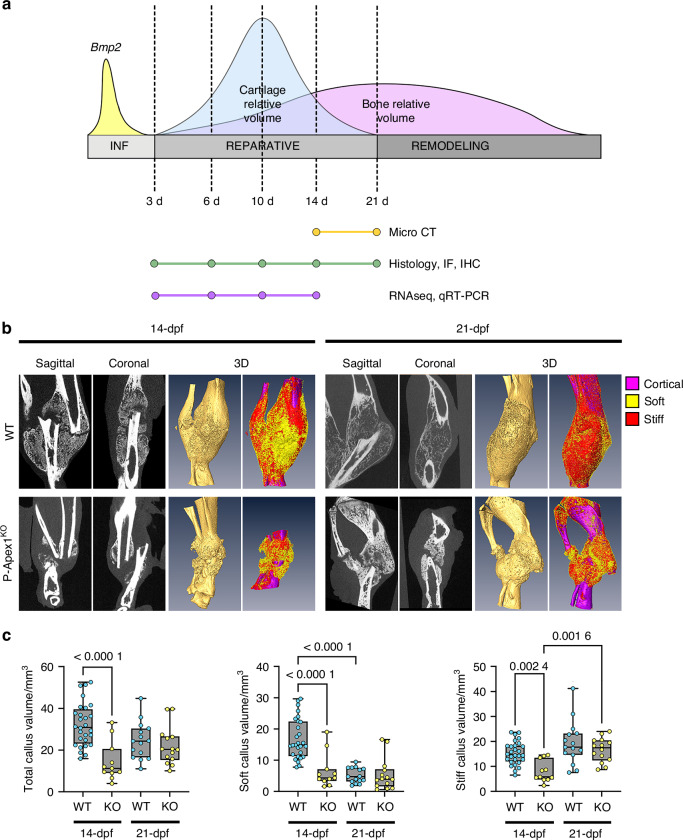
Silencing *Apex1* in limb bud mesenchyme delays fracture healing causing fracture nonunion. **a** Graphical representation of the fracture healing phases, the relative volumes for bone and cartilage and the experimental design for the analysis of the effects of *Apex1* silencing. **b** Representative radiographic planes, 3D reconstruction and callus segmentation for WT and P-Apex1^KO^ mice at 14- (WT, *n* = 28; P-Apex1^KO^, *n* = 11) and 21-dpf (WT, *n* = 15; P-Apex1^KO^, *n* = 14). **c** Quantification of the total callus volume, soft tissue (non-mineralized), and stiff tissue (mineralized) callus volume. Results are expressed as median with interquartile range; whiskers represent maximum and minimum values. *P* values were determined by one way ANOVA (Total callus volume, *P* < 0.000 1; Soft callus volume, *P* < 0.000 1; Stiff callus volume, *P* < 0.000 1) followed by Sidàk’s multiple comparisons test

Once we determined that *Apex1* silencing does not cause mayor morphological alterations in the diaphysis of adult mice limbs, we evaluated the role of *Apex1* in the fracture repair process. For this purpose, we performed a non-stabilized tibia fracture in P-Apex1^KO^ mice and WT littermates. The reparative process was allowed to proceed for a maximum of 21 days (21-dpf), and the fractures were analyzed at different time points of the reparative phase with specific analysis, as represented in Fig. 2a.^9,36–39^ Initial radiographical analysis was carried out by μCT at 14- and 21-dpf, three-dimensional reconstruction of the fracture callus showed that, at 14-dpf, WT mice presented a well-developed callus with a radiolucent fracture line, achieving bone union at 21-dpf. In P-Apex1^KO^ mice, at 14-dpf, we detected an underdeveloped fracture callus with a wide radiolucent area and reduced bone content, localized at the distal regions of the fracture line. By 21-dpf, P-Apex1^KO^ mice showed an increased bone content, but with a persistent radiolucent fracture line and sclerotic bone in 7 out of 14 animals (Fig. 2b). Quantitative analysis showed that at 14-dpf P-Apex1^KO^ mice presented a significant reduction in the total volume of the callus, showing a marked decrease of both soft and stiff tissues. Interestingly, these differences disappeared at 21-dpf. In WT mice, we noted a significant reduction in the volume of non-mineralized tissue, whereas this process was impaired in P-Apex1^KO^ mice (Fig. 2c).

The radiological and morphometric analysis of the fracture callus suggests that APEX1 participates in at least two key processes of the fracture healing. The reduced callus volume at 14-dpf suggest that the initial response to bone injury is impaired or delayed when *Apex1* is silenced, setting APEX1 functions in mesenchymal progenitor cells. Furthermore, the radiographic and volumetric data point to compromised cartilage resorption, which disrupts healing progression in P-Apex1^KO^ mice, positioning the role of APEX1 in chondrocyte differentiation, as noted during appendicular skeleton development.

### Apex1 silencing impairs fracture healing initiation

**Fig. 3:**
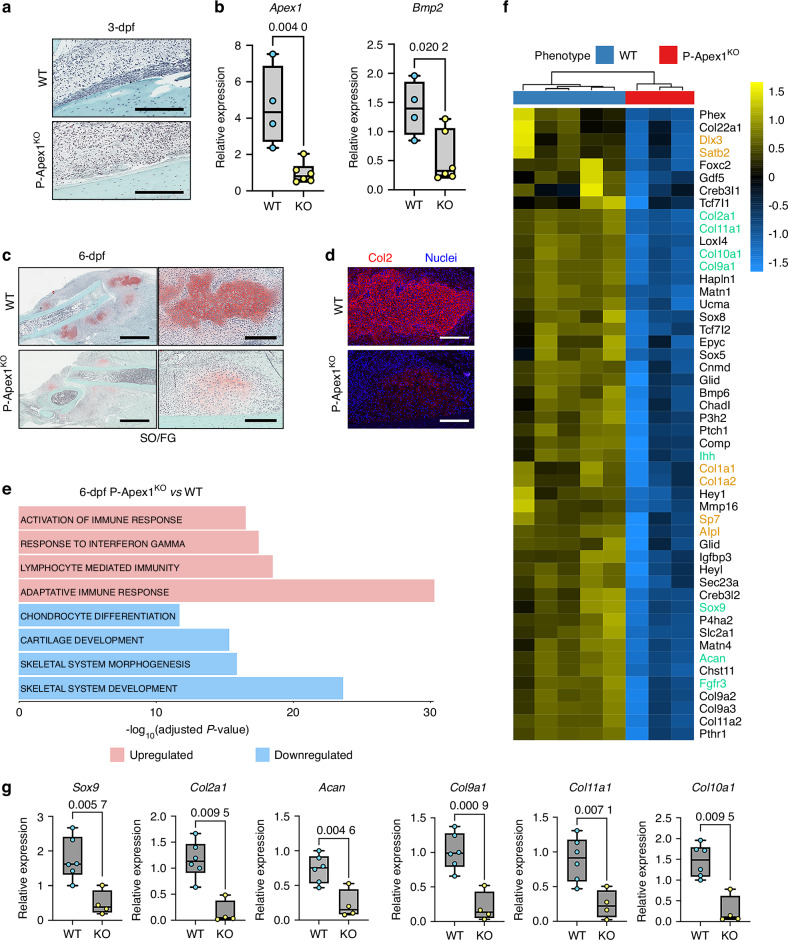
*Apex1* silencing impairs fracture healing initiation. **a** Histological analysis of the fracture callus stained with Safranin O/Fast Green at 3-days post-fracture (dpf). Scale bar 300 μm. **b** Expression levels for *Apex1* and *Bmp2* in 3-dpf fracture callus (WT, *n* = 4; P-Apex1^KO^, *n* = 6). *P* values were determined by unpaired Student’s t-test. **c** Histological analysis of the fracture callus stained with Safranin O/Fast Green at 6-dpf. Scale bar, left panels 1.5 mm; right panels, 300 μm. **d** Immunohistological detection of the content of type II collagen (COL2) in the 6-dpf fracture callus. **e** Representation of the top upregulated (red columns) and downregulated (blue columns) GO Biological processes from a gene set enrichment analysis. **f** Heatmap showing the scaled expression of genes related to endochondral ossification and chondrogenesis that are significantly downregulated in P-Apex1^KO^ fracture callus at 6-dpf (WT, *n* = 5; P-Apex1^KO^, *n* = 3). Highlight in orange, essential osteogenic markers; highlight in green, key chondrogenic markers. **g** qPCR analysis for chondrogenic markers validating the DEGs at 6-dpf. Results are expressed as median and interquartile range; whiskers represent maximum and minimum values. WT, *n* = 6; P-Apex1^KO^, *n* = 4. *P* values were determined by unpaired Student’s t-test (*Sox9*, *Acan*, *Col9a1*, *Col11a1*) or Mann-Whitney test (*Col2a1*, *Col10a*1)

To better understand the impact of *Apex1* silencing during the initial healing response, we analyzed histologically the fracture callus at 3-dpf, a time point marking the end of the inflammatory phase and the beginning of the reparative phase. In WT mice, we observed histological signs of an initial fracture healing response, including an expanded periosteal layer in the vicinity of the fracture site.^11^ However, in P-Apex1^KO^ mice, this periosteal response was either absent or delayed (Fig. 3a). In addition, quantitative RT-PCR of the fracture zone showed that the expression of *Bmp2* was significantly impaired when compared with WT littermates, suggesting a direct effect of APEX1 in the fracture healing initiation through the transcriptional control of *Bmp2* (Fig. 3b).

The impact of *Apex1* silencing on periosteal cell proliferation was confirmed in WT and P-Apex1^KO^ mice using immunohistochemical staining against Ki67, a nuclear marker of active cell proliferation (Fig. S4a).^40,41^

To gain insight into the molecular effects of *Apex1* silencing at 3-dpf, fractured tibiae were dissected around the fracture site, and total RNA was isolated and subjected to bulk RNA sequencing (RNA-seq). Gene set enrichment analysis (GSEA) revealed that *Apex1* silencing significantly downregulated the smoothened signaling pathway, along with processes related to cell division and DNA repair, sister chromatid segregation or regulation of chromosome segregation, confirming an impairment of proper initial callus formation.^42,43^ Conversely, *Apex1* silencing led to the enrichment of processes related to muscle activity and metabolism, including striated muscle cell development, sarcomere organization, and muscle contraction (Fig. S4b, Table S1). Overall, although statistically significant, these changes were subtle, and no major differentially expressed genes (DEGs) were identified (Fig. S4c, Table S2).

Next, we evaluated fracture healing progression at 6-dpf, a time point in the early reparative phase of fracture healing. Histological analysis showed that WT mice presented an incipient fracture callus containing abundant cartilage tissue (Safranin O positive areas) and reduced new bone areas at the distal edges of the fracture line. In P-Apex1^KO^ mice, fracture calluses were smaller, containing less cartilage and bone tissues compared to WT mice calluses (Fig. 3c, Fig. S5a). The differences in cartilage content were confirmed by immunofluorescence against type II collagen. As expected, WT mouse cartilage areas were labeled with an intense reaction to type II collagen. In P-Apex1^KO^ mice, there was an important reduction in the extension and intensity of the labeling for type II collagen, suggesting an impaired chondrogenic response after injury (Fig. 3d).

To further clarify the mechanisms implicated in the silencing of *Apex1* during fracture repair, we performed a transcriptomic analysis of 6-dpf calluses in WT and P-Apex1^KO^ mice. Whole callus bulk RNA-seq analysis showed that P-Apex1^KO^ mice displayed an altered transcriptomic profile with 1 715 genes significantly downregulated and 1 614 genes significantly upregulated (Fig. S5b). A gene set enrichment analysis (GSEA) showed downregulation of crucial processes related to endochondral ossification after *Apex1* silencing, including chondrocyte differentiation, cartilage development, skeletal system morphogenesis, and skeletal system development. Interestingly, the biological processes related to the activation of the immune response, response to interferon gamma, lymphocyte mediated immunity, and adaptive immune response were upregulated (Fig. 3e, Table S1).

In addition, Hedgehog and TGF-β/BMP were the major downregulated signaling pathways at this time point (Fig. S5c, Table S1).

Among the list of downregulated genes, key players were identified implicated in different stages of endochondral ossification, chondrocyte differentiation and cartilage maturation (*Sox9*, *Acan*, *Col2a1*, *Col9a1*, *Col10a1*, *Col11a1*, Fgfr3, *Ihh*), as well as osteoblasts differentiation (*Sp7*, *Alpl*, Satb2, *Dlx3*, *Col1a1*, *Col1a2*), confirming the impaired bone regeneration response in P-Apex1^KO^ mice (Fig. 3f, Table S2). The impaired chondrogenesis response in P-Apex1^KO^ was further validated by qPCR in total RNA extracted from whole 6-dpf calluses (Fig. 3g).

Histological, immunohistological and transcriptional data collectively support the notion that *Apex1* plays a key role in the initiation of the fracture healing process.

### Apex1 silencing impairs fracture healing progression

To establish the mechanisms affected by *Apex1* silencing during the late reparative phase, we conducted a histomorphometric analysis of the cartilaginous tissue. Fracture callus from WT and P-Apex1^KO^ was collected at 10-, 14-, and 21-dpf, stained for Safranin O/Fast Green, and assessed for cartilage tissue content.

**Fig. 4:**
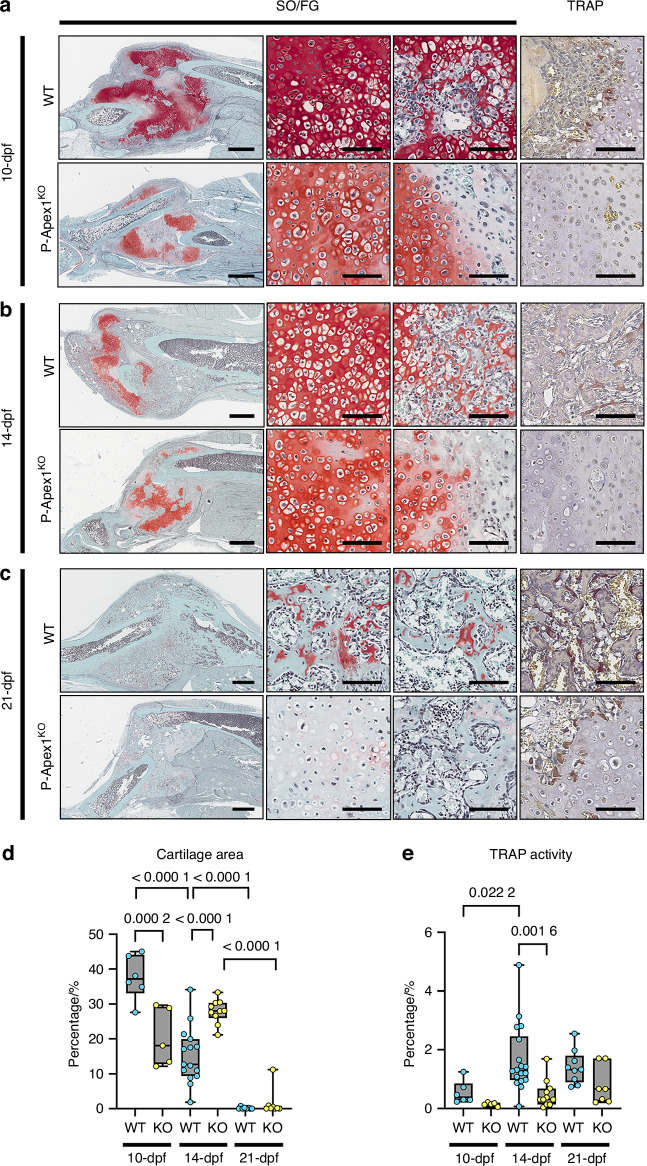
*Apex1* silencing impairs fracture healing progression. Histological analysis at 10-dpf (**a**), 14-dpf (**b**), and 21-dpf (**c**). SO/FG, Safranin O/Fast green. TRAP, tartrate resistant acid phosphatase staining. Left panels, whole callus view; middle left panels, cartilage view; middle right panels, cartilage-bone interphase; right panels, TRAP staining of the cartilage bone interphase. Scale bar, left panel, 1 mm; middle and right panels, 100 μm. Arrowheads show the presence of TRAP^+^ cells. **d** Histomorphometric quantification of the cartilage area at 10-dpf (WT, *n* = 6; P-Apex1^KO^, *n* = 5), 14-dpf (WT, *n* = 15; P-Apex1^KO^, *n* = 10), and 21-dpf (WT, *n* = 8; P-Apex1^KO^, *n* = 8). Percentage/%, cartilage area/total callus area x 100. Results are expressed as median and interquartile range; whiskers represent maximum and minimum values. *P* values were determined by one way ANOVA (*P* < 0.000 1) followed by a Sidàk’s multiple comparisons test. **e** Histomorphometric quantification of TRAP activity at 10-dpf (WT, *n* = 6; P-Apex1^KO^, *n* = 5), 14-dpf (WT, *n* = 18; P-Apex1^KO^, *n* = 11), and 21-dpf (WT, *n* = 9; P-Apex1^KO^, *n* = 7). Percentage/%, TRAP positive area/Total callus area x 100. Results are expressed as median and interquartile range; whiskers represent maximum and minimum values. *P* values were determined by one way ANOVA (*P* = 0.000 2) and Sidàk’s multiple comparisons test

At 10-dpf, WT calluses consisted of cartilage in different stages of maturation, ranging from proliferative to hypertrophic, as well as distinctive bone areas progressing through the cartilaginous tissue, where degradation of cartilage was evident (Fig. 4a, central panels). These areas of cartilage degradation were positive for TRAP staining, indicating active remodeling (Fig. 4a, right panel). In P-Apex1^KO^ calluses, the total cartilage area was reduced, but since the overall callus area was also smaller, the cartilage remained the dominant component of the callus. Notably, chondrocyte morphology in P-Apex1^KO^ mice appeared in a prehypertrophic stage, with no evidence of cartilage degradation or TRAP positive cells at the edges of the cartilage areas (Fig. 4a).

At 14-dpf, WT calluses showed an increase in bone tissue and a notable reduction of the cartilaginous areas, with more pronounced regions of cartilage degradation/resorption compared to 10-dpf. P-Apex1^KO^ calluses displayed a cartilage content similar to that at 10-dpf, despite some increase in bone content. No signs of degradation/resorption were yet present at the cartilage islands of P-Apex1^KO^ calluses (Fig. 4b).

At 21-dpf, WT calluses exhibited only traces of cartilage resorption, integrated in the new trabecular bone of the callus. In P-Apex1^KO^ calluses, the safranin positive areas had faded, but cartilage regions could still be identified by the morphology of the cells and type II collagen immunohistochemistry (Fig. S6). In P-Apex1^KO^ calluses, TRAP-positive cells were observed at the border of the cartilage islands and on the surface of the new trabecular bone (Fig. 4c).

To better illustrate the differences in callus maturation between WT and P-Apex1^KO^ mice, we performed a histomorphometric quantification of cartilaginous tissue (Safranin O positive, SO) and TRAP activity (TRAP staining) at 10-, 14- and 21-dpf. Quantification of the cartilaginous tissue demonstrated that WT calluses contained significantly more cartilage than P-Apex1^KO^ calluses. However, while WT calluses showed a lineal and significant reduction in cartilage content between 10-, 14-, and 21-dpf, the reduction in P-Apex1^KO^ calluses was only detected between 14-dpf to 21-dpf, likely due to reduced SO staining rather than true cartilage resorption. Notably, at 14-dpf, P-Apex1^KO^ calluses had a significantly higher percentage of cartilage, further supporting the lack of cartilage resorptive areas at 10- and 14-dpf (Fig. 4d). TRAP activity quantification concurred with these observations, showing that while TRAP activity peaked at 14-dpf in WT calluses, it remained low in P-Apex1^KO^ until increasing at 21-dpf (Fig. 4e).

To further characterize the processes and molecular pathways affected by *Apex1* silencing, we performed bulk RNA-seq analysis of the fracture calluses from WT and P-Apex1^KO^ mice at 10-dpf (end of the soft callus formation), and 14-dpf (peak of cartilage resorption).

Differential expression analysis revealed significant transcriptomic differences in P-Apex1^KO^ mice at both time points, with 2 930 differentially expressed genes at 10-dpf (1 288 downregulated, 1 092 upregulated) and 6 467 differentially expressed genes at 14-dpf (3 184 downregulated, 3 283 upregulated) (Fig. S7a, b).

**Fig. 5:**
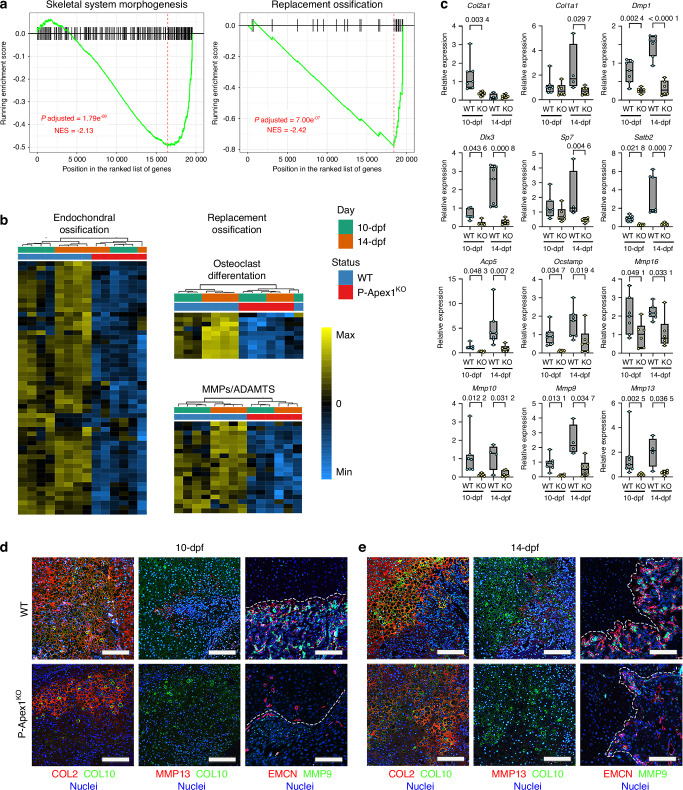
*Apex1* silencing in limb bud mesenchyme impairs terminal chondrocyte differentiation. **a** Gene set enrichment analysis (GSEA) plots showing the impact of *Apex1* silencing on key processes for endochondral ossification progression at 14-dpf. **b** Heatmaps representing the scaled expression of genes implicated in different aspects of endochondral ossification downregulated at 10- and 14-dpf due to *Apex1* silencing. **c** Quantitative RT-PCR analysis validating the bulk RNA-seq data, all results are expressed as median and interquartile range; whiskers represent maximum and minimum values. 10-dpf (WT, *n* = 7; P-Apex1^KO^, *n* = 6), 14-dpf (WT, *n* = 4-7; P-Apex1^KO^, *n* = 6). *P* values were determined by one way ANOVA (*Col2a1*, *P* = 0.001 1; *Dmp1*, *P* < 0.000 1; *Ocstamp*, *P* = 0.001 8; *Mmp16*, *P* = 0.016 6) or Kruskal Wallis test (*Col1a1*, *P* = 0.069 4; *Dlx3*, *P* = 0.000 1; *Sp7*, *P* = 0.006 2; *Satb2*, *P* < 0.000 1; *Acp5*, *P* = 0.000 3; *Mmp9*, *P* = 0.000 9; *Mmp10*, *P* = 0.003 4; *Mmp1*3, *P* = 0.00 1) followed by Sidàk’s or Dunn’s multiple comparisons tests. **d** Immunohistological detection of markers of cartilage maturation and endochondral ossification progression for WT and P-Apex1^KO^ at 10-dpf (**f**) and 14-dpf (**e**). Dotted white lines label the chondro-osseous junction of the fracture callus. Scale bar, 100 μm

GSEA indicated downregulation of crucial processes involved in endochondral ossification progression at 10- and 14-dpf in P-Apex1^KO^ calluses, including skeletal system morphogenesis, and replacement ossification (Fig. 5a, Fig. S7c). Upregulated processes in P-Apex1^KO^ mice were predominantly related to the adaptative immune system, such as the adaptative immune response and lymphocyte mediated immunity (Fig. S7d).

Differential gene expression showed a global impairment in endochondral ossification progression in P-Apex1^KO^ calluses, particularly affecting genes involved in endochondral ossification, osteoclast differentiation, and genes codifying for MMPs and ADAMTS (Fig. 5b, Fig. S8, Table S1). The RNA-seq results were validated using quantitative RT-PCR for key endochondral ossification markers. In WT mice, there was a clear progression from cartilage to bone in the fracture callus, shown by a decrease in the expression of *Col2a1* and an increase in *Col1a1* and *Dmp1*, both essential components of the extracellular matrix (ECM), as well as transcription and nuclear factors mediating osteoblast differentiation and ECM calcification (*Dlx3*, *Sp7*, *Satb2*). In contrast, the ossification process was disrupted in P-Apex1^KO^ mice. Quantitative RT-PCR showed that genes needed for osteoclasts differentiation (*Acp5*, *Ocstamp*), and MMPs responsible for breaking down cartilaginous ECM (*Mmp16*, *Mmp10*, *Mmp13*, *Mmp9*), were significantly downregulated (Fig. 5c, Table S2).

Additionally, we performed GSEA for the broader Hallmark gene sets looking for relevant signaling pathways and their associated DEG. The TGFβ/BMP, NOTCH, and WNT signaling pathways were significantly downregulated at 10-dpf and 14-dpf in P-Apex1^KO^ mice (Fig. S9, Table S1). DEG belonging to the TGF-β/BMP and NOTCH and WNT signaling pathway were identified as consistently downregulated in P-Apex1^KO^ animals (Fig. S10a, Table S2). The downregulation of key transcription factors within these pathways was further confirmed by quantitative RT-PCR (Fig. S10b).

Immunohistochemical analysis corroborated these transcriptomic findings. ECM maturation was analyzed by the presence of type II and type X collagen. In WT mice at 10-dpf, abundant ECM deposition was observed, positive for type II and type X collagen, with areas of cartilage degradation defining chondro-osseus-like edges that were positive for MMP-13 and MMP-9, and associated with important vascular invasion represented by type H vessels (Endomucin positive cells). In contrast, P-Apex1^KO^ mice displayed sparse ECM deposition, diminished type X collagen, undetectable levels of MMP-13 and MMP-9, and reduced Endomucin positive cells, indicating impaired vascular invasion (Fig. 5d). At 14-dpf, WT calluses showed an increased cartilage resorption and trabecular bone formation, with enhanced MMP-9 and MMP-13 and Endomucin presence, outlining the progression of the endochondral ossification process and fracture repair. P-Apex1^KO^ mice, however, exhibited no significant changes in cartilage maturation, ECM resorption, presence of MMP-13, MMP-9, and vascular invasion, confirming the impaired endochondral and replacement ossification processes (Fig. 5e).

Altogether, the transcriptomic and immunohistological analysis indicate that the impaired maturation of the chondrocytes at the end of the reparative phase determines the phenotype observed in P-Apex1^KO^ fracture calluses.

### APEX1 functions are needed in mesenchymal progenitors during fracture healing initiation

**Fig. 6:**
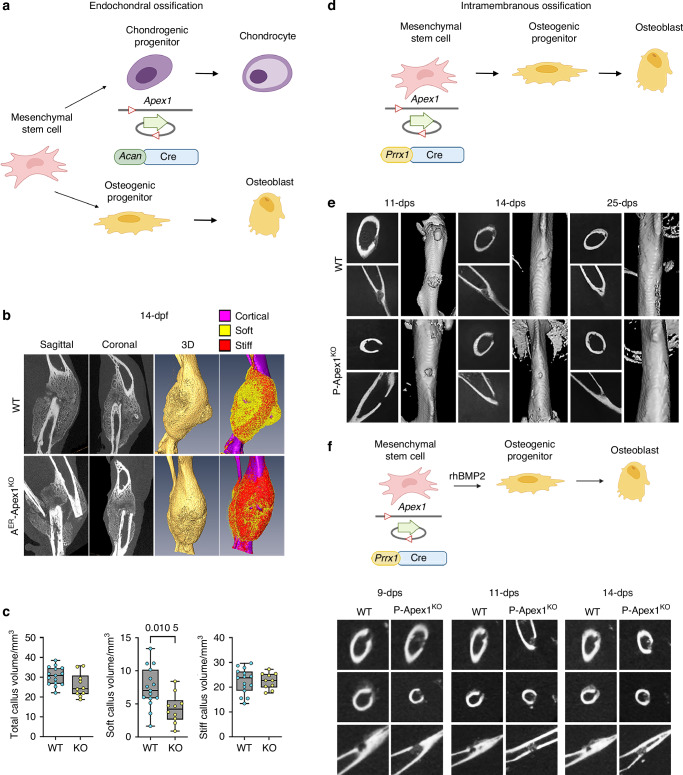
APEX1 function is needed in mesenchymal progenitor cells during the inflammatory phase of fracture healing. **a** Genetic strategy for Apex1 silencing in chondrocytes during endochondral ossification using Acan Cre deleter. **b** Representative radiographic planes, 3D reconstruction, and segmentation at 14-dpf for WT (*n* = 15) and A^ER^-Apex1^KO^ (*n* = 10). **c** Quantification of the total, soft (nonmineralized) and stiff (mineralized) callus volume at 14-dpf. Results are expressed as median and interquartile range; whiskers represent maximum and minimum values. *P* values determined by two-tailed Student’s t test. **d** Genetic strategy for Apex1 silencing during intramembranous ossification. **e** Representative radiographic planes and 3D reconstruction of femoral diaphysis at 11-, 14- and 25-days post-surgery (-dps) of WT (11-dps, *n* = 5; 14-dps, *n* = 5; 25-dps, *n* = 2), and P-Apex1^KO^ (11-dps, *n* = 5; 14-dps, *n* = 7; 25-dps, *n* = 4) mice that received a subcritical (0.8 mm) monocortical bone defect. Yellow arrow indicates the position of the monocortical defect. **f** Upper panel, rescue strategy for the phenotype of *Apex1* silencing during intramembranous ossification. Lower panels, representative radiographical planes of the femoral diaphysis at 9-, 11-, and 14-dps of WT (*n* = 3 each time point) and P-Apex1^KO^ (*n* = 3 each time point) that received a subcritical unicortical bone defect and an implant of absorbable collagen sponge containing 100 ng of rhBMP-2. Yellow arrowheads indicate the position of the monocortical defects

To better define the cellular mechanisms affected by APEX1 during fracture healing, we asked whether the observed fracture phenotype was derived from the chondrocyte-impaired hypertrophic process. We hypothesized that if the nonunion phenotype observed in Prrx1-driven silencing was due exclusively to APEX1 functions in chondrocytes, silencing *Apex1* only in chondrocytes would reproduce the observed phenotypes (Fig. 6a). We sought to silence *Apex1* during the reparative phase in chondrocytes, starting after 2-dpf, using aggrecan promoter (Acan-CreER^T2^). After verifying the chondrocytes as the target population for Acan-CreER^T2^ mediated silencing (Fig. S11), we crossed Acan-CreER^T2^;Apex^C/+^ mice with Apex1^C/C^ mice. The resulting Acan-CreER^T2^;Apex1^C/C^ (A^ER^-Apex1^KO^) and WT littermates (Acan-CreER^T2^;Apex1^C/+^, Apex1^C/C^, Apex1^C/+^) were subjected to a closed tibia fracture. All animals received tamoxifen injection at 2-, 4- and 6-dpf and were sacrificed at 14-dpf, the time at which the P-Apex1^KO^ fracture phenotype presented 100% penetrance. At 14-dpf, radiographic and three-dimensional reconstruction of the callus showed that both A^ER^-Apex1^KO^ and WT littermates showed a well-developed callus without major radiological differences (Fig. 6b). Morphometric analysis revealed that there were no significant differences in the total callus volume or the mineralized tissue content between the two groups, although A^ER^-Apex1^KO^ mice presented a significant reduction in the volume of nonmineralized tissue (WT, 7.449 ± 3.088; A^ER^-Apex1^KO^, 4.282 ± 2.234, *P* = 0.010 5) (Fig. 6c).

Histological assessment of SO/FG-stained calluses at 10-dpf and 14-dpf in both WT and A^ER^-Apex1^KO^ calluses revealed a reduced cartilage content in A^ER^-Apex1^KO^ mice at 10-dpf. At 14-dpf, cartilage area reduction was evident in both groups (Fig. S12a). Interestingly, resorption areas with positive TRAP staining could be identified in both groups at 10- and 14-dpf with no major differences (Fig. S12b, c).

Immunodetection of chondrocyte maturity markers (type II and type X collagen), matrix metalloproteinases (MMP-13 and MMP-9), and vascular invasion showed that the expression of hypertrophic markers (type X collagen and MMP-13) in A^ER^-Apex1^KO^ mice was reduced or absent in cartilage resorption areas at both 10- and 14-dpf. In addition, the expression of MMP-9 was also impaired in the invading vasculature, though the signal for Endomucin was similar between WT and A^ER^-Apex1^KO^ mice (Fig. S11d, e).

Interestingly, silencing *Apex1* at the end of the inflammatory phase impacted the formation of the non-mineralized callus but did not affect the final properties of the mineralized callus. We conclude that APEX1 is required in progenitor mesenchymal cells, and silencing *Apex1* only in committed chondroprogenitors has a minor effect on the global fracture healing process.

Additionally, to determine if APEX1 has a role in intramembranous ossification, we created a 0.8 mm monocortical defect in the femur of P-Apex1^KO^ and WT littermates that directed bone repair through intramembranous ossification (Fig. 6d). We monitored bone repair progression at 11-, 14-, and 25- days post-surgery (-dps) and found that the healing response in P-Apex1^KO^ mice was delayed at 11- and 14-dps. Remarkably, although the defect was also almost totally covered at 25-dps in P-Apex1^KO^ mice, a periosteal reaction was not observed at any of the time points analyzed (Fig. 6e).

All together, these results suggest *Apex1* silencing in mesenchymal progenitors impacts the formation of the fracture callus during the inflammatory phase, affecting both endochondral and intramembranous ossification-mediated healing.

### Silencing Apex1 impairs Bmp2 expression during the inflammatory phase

At the molecular level, fracture healing is initiated by the expression of *Bmp2/*BMP2 during the inflammatory phase, peaking 24 h post fracture.^9,17^ Silencing *Bmp2* in limb mesenchyme or disturbing *Bmp2* expression during the inflammatory phase results in a nonunion-like phenotype.^11,17^ We hypothesized that *Apex1* silencing in mesenchymal progenitors impairs *Bmp2* expression peak during the inflammatory phase, delaying fracture healing initiation and producing a small callus through hindered chondrogenesis and osteogenesis. To validate this hypothesis, we reasoned that overexpressing or adding exogenous BMP-2 would rescue the observed phenotypes.

To determine the impact of *Bmp2* overexpression during fracture healing that progresses through endochondral ossification, we designed an experiment to overexpress *Bmp2* in mice (Prrx1CreER^T2^;ROSA26^Tg-Bmp2-IRES-EGFP^, P^ER^-TgBmp2). *Bmp2* overexpression was induced immediately after bone fracture by tamoxifen injection on days 0, 1 and 2 post-fracture. The effect of this overexpression on fracture repair was analyzed morphometrically at 14- and 21-dpf by μCT. Interestingly, *Bmp2* overexpression during the inflammatory phase significantly increased the total callus and soft callus volumes at 14-dpf. By 21-dpf, there were no differences in callus morphometric properties between the groups, indicating that fracture healing progression mechanisms were not affected by the overexpression of *Bmp2* (Fig. S13).

To determine the impact of BMP2 overexpression during fracture healing that progresses through intramembranous ossification, we performed a mono cortical defect in WT and P-Apex1^KO^ animals implanting an absorbable collagen sponge containing 100 ng of rhBMP-2. Adding exogenous rhBMP-2 rescued the phenotype observed during intramembranous ossification mediated repair and no differences between WT and P-Apex1^KO^ mice were detected as early as 9-dps (Fig. 6f).

**Fig. 7:**
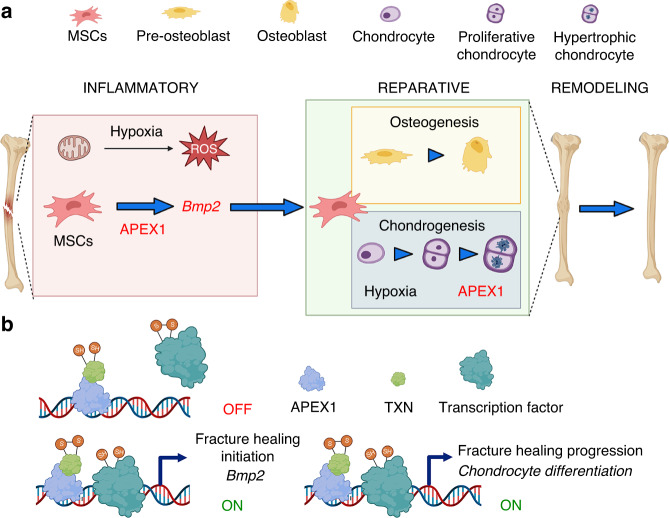
Cellular and molecular mechanism of APEX1 function during fracture healing. **a** APEX1 function is needed in mesenchymal progenitors during the inflammatory phase to an efficient expression of *Bmp2* and in chondrocytes during the reparative phase driving chondrocyte differentiation for a proper fracture healing progression. **b** Suggested molecular mechanism of APEX1 function during fracture healing. APEX1 together with Thioredoxin (TXN) allows the reduction of specific cysteines during transcription factors DNA binding and final activation. During the inflammatory phase APEX1 and TXN activate transcription factors controlling *Bmp2* expression in mesenchymal progenitor cells. During the reparative phase APEX1 and TXN activate transcription factors controlling genes driving the chondrocyte differentiation and fracture healing progression

All together, we concluded that APEX1 activities during the inflammatory phase control the initial reparative response and the fracture callus volume through the control of the *Bmp2* expression level in mesenchymal progenitor cells. In addition, APEX1 activity is needed in the progression of endochondral ossification by the control of chondrocyte differentiation (Fig. 7).

## Discussion

In this study, we provide genetic evidence of the pivotal role that ROS signaling plays in chondrocyte differentiation, endochondral ossification progression and fracture healing initiation. Our findings demonstrate that APEX1 function is crucial to these processes and uncovers a molecular pathway whose disruption could induce the appearance of fracture nonunion. In addition, we demonstrate that fracture nonunion is defined by impaired APEX1 mediated signaling during the inflammatory phase.

APEX1 is a multifunctional protein with two well-established functions, DNA repair and redox regulation of transcription factors, positioning it at the core of the cellular response to ROS and oxidative stress.^44,45^ Although both APEX1 activities are suppressed in our model, we propose that the redox function underlies the observed phenotypes. Post-translational modifications involving cysteine oxidation are known to regulate the DNA binding and transcriptional activity of key transcription factors, such as AP-1 (FOS/JUN heterodimer), HIF-1α, or the CREB family, all of which have been identified as promoters of chondrocyte hypertrophy.^46–49^ Notably, AP-1 plays a crucial role controlling the expression of MMPs during physiological and pathological conditions, while HIF-1 controls VEGF expression in chondrocytes.^50–52^

Our current findings, combined with previous experimental evidence, underscore the crucial interplay between oxidative stress and *Bmp2* expression during fracture healing, identifying ROS imbalance as a key determinant of fracture nonunion. By abolishing the TXN redox-scavenging function using PX-12, we showed that the resulting oxidative stress impaired *BMP2* expression in human periosteum explants and induced a nonunion-like phenotype in fractured mice. Strikingly, inhibition of *Bmp2* expression and subsequent fracture nonunion appearance occurred only when PX-12 was administered during the inflammatory phase, not after *Bmp2* expression peak or during the reparative phase.^17^ It is well-established that the expression of *Bmp2* immediately after injury is needed for fracture healing initiation.^10,53^ Our current findings support the hypothesis that APEX1 regulates *Bmp2* expression during the early stages of fracture healing. Silencing *Apex1* mirrors the effect of *Bmp2* silencing in mesenchymal progenitors, leading to an absence of periosteal response, impaired chondrocyte and osteoblast differentiation, and a failure to revascularize the fracture callus. Conversely, overexpressing *Bmp2* during fracture healing significantly increases cartilage synthesis during the reparative phase. Taken together, both results suggest that the extent of the reparative response is proportional to *Bmp2* expression levels during the inflammatory phase (Fig. S13).

Interestingly, silencing *Bmp2* in chondrocytes produced a milder effect on fracture healing, and no significant impact was observed when *Bmp2* was specifically silenced in osteoblasts or endothelial cells.^54,55^ Similarly, *Apex1* silencing in limb bud mesenchyme has the greatest impact on fracture healing, whereas its silencing in chondrocytes has a more limited effect, despite the presence of impaired chondrocyte differentiation. Thus, our results suggest that APEX1 has an important role in the expression of *Bmp2*, though other factors are sure to contribute to its transcriptional control, as *Bmp2* expression is not completely abolished and a blunted callus still develops in P-Apex1^KO^ animals.

During development, silencing *Apex1* in limb mesenchyme impairs chondrocyte differentiation, resulting in a phenocopy of *Mmp9*, *Mmp13* single knockout and more evidently *Mmp9*/*Mmp13* double knockout models.^56–58^ In humans, mutations of *MMP13* or *MMP9* result in metaphyseal anadysplasia, a metaphyseal dysplasia that resolves with age. Likewise, in mice, growth plate abnormalities from *Mmp9* or *Mmp13* silencing also resolve with age.^59^ Our data reveals *Apex1* silencing reproduces these mutations, with impaired terminal chondrocyte differentiation leading to reduced expression of type X collagen and *Mmp13*. Unlike the phenotype observed in *Mmp13* knockout mice, where chondrocyte differentiation is unaffected and the morphology, number, and vasculature location seem normal, P-Apex1^KO^ mice exhibit disrupted vasculature.^56^ This phenotype suggests *Apex1* silencing blocks chondrocyte differentiation, halting chondrocytes in a prehypertrophic state. Hypertrophic chondrocytes are known to secrete type X collagen, MMPs (MMP-13), angiogenic factors (VEGF), and chemokines and cytokines (RANKL) to attract monocyte/macrophages and induce their differentiation into chondroclasts/osteoclasts, facilitating the removal of the avascular cartilage and the invasion of blood vessels and osteoblasts.^47,60–62^ In *Mmp13* or *Mmp9*-silenced mice, all these angiogenic and differentiation factors remain present in the ECM, aiding eventual cartilage removal.

During fracture healing, *Apex1* silencing also impacts the chondrocyte differentiation, delaying cartilage scaffold resorption and fracture healing progression. We detected a significant reduction in the number of TRAP positive cells, reduced expression of MMP-13 and MMP-9, and limited vascular invasion. Silencing of *Mmp13*, *Mmp9* or *Mmp1*0 results in hypertrophic cartilage accumulation maintaining an elevated callus volume at the end of the reparative phase (10-, 14-, 21- and even at 28-dpf), though healing is ultimately accomplished.^13,15,16^ However, since *Apex1* silencing also impairs fracture healing initiation, we did not observe the callus enlargement characteristic of the early reparative phase, nor the subsequent reduction in callus volume typically observed in WT littermates during the latter reparative phase. Interestingly, *Apex1* specific silencing in chondrocytes (A^ER^-Apex1^KO^) cannot reproduce the phenotype observed when *Apex1* is silenced in limb bud mesenchyme (P-Apex1^KO^). However, our experimental data indicate that the chondrocyte phenotype is present, with no MMP-13 or type X collagen presence in the cartilage intermediate, suggesting a more complex interplay of factors. A possible explanation is that *Apex1* plays no role when *Mmp13* is expressed exclusively by the osteoblasts. In fact, while cartilage is avascular and chondrocytes exist in a hypoxic environment, bone tissue and osteoblast differentiation are highly dependent on oxygen tension. Therefore, the expression of *Apex1* and/or the functions of APEX1 may differ between chondrocytes and osteoblasts.^63,64^

Transcriptomic analysis revealed that *Apex1* silencing impacts key signaling pathways during fracture repair throughout the healing process. At the end of the inflammatory phases (3-dpf), GSEA identified smoothened/hedgehog (Hh) signaling as one of the major downregulated pathways. The Hh signaling pathway has been shown to be critical for the commitment of mesenchymal progenitor cells to chondrocytes, and loss of the *Smo* gene in *Sox9*^+^ periosteal cells resulted in near-complete failure of cartilage callus formation, a phenotype closely resembling P-Apex1^KO^ mice.^65^ Additional experimental data would be necessary to determine the interdependence between the expression of Hh signaling effectors, i.e., *Shh*, and *Bmp2*. In any case, this downregulation persisted into the early reparative phase (6-dpf), confirming that the impaired chondrogenesis observed in P-Apex1^KO^ mice is defined during the inflammatory phase. At this stage, GSEA revealed impaired response to BMP and downregulation of major signaling pathways that have been demonstrated to be fundamental for endochondral ossification such as WNT and NOTCH, implicated in tissue injury response and chondrocyte proliferation^66^; osteoblastogenesis^67^; and chondrocyte maturation to hypertrophy, as well as vascular and osteogenic invasion of the hypertrophic cartilage.^68^ Finally, at the end of the reparative phase (10- and 14-dpf), BMP, NOTCH, and WNT, key signaling pathways that participate in chondrocyte maturation are the major pathways affected by *Apex1* silencing. On one hand, BMP signaling is involved in most processes related with endochondral ossification during skeletal development. It regulates the expression of *Sox9*, which controls the commitment of mesenchymal progenitor cells into chondrocytes, and *Runx2*, which plays a central role in the differentiation of mesenchymal progenitor cells into osteoblasts, but also in the maturation of proliferative chondrocytes into hypertrophic chondrocytes.^69^ Additionally, BMP has been reported to upregulate NOTCH and WNT signaling, further promoting chondrocyte differentiation.^70^

In conclusion, we propose a model for fracture healing initiation that is dependent on *Bmp2* expression in mesenchymal progenitor cells. Immediately after bone injury, hypoxia initiates a signaling pathway mediated by reactive oxygen species (ROS) that modifies the oxidative status of cysteine containing proteins. In this signaling pathway TXN and APEX1 act as the final transducers of key transcription factors controlling significant genes, including *Bmp2* and/or *Shh*, that determine the starting of the reparative response. In addition, our results demonstrated that APEX1 also controls essential transcription factors implicated in the terminal differentiation of chondrocytes and drive fracture healing progression.

## MATERIALS AND METHODS

### Mice

All animal procedures were approved by the University of Navarra Institutional Committee on Care and Use of Laboratory Animals (CEEA) and the Navarra Regional Government (CEEA #106-23, #E28-18(105-17E1), #E40-18(105-17E2), #E40-19(105-17E5), #E21-20 (105-17E6).

Mouse strains, B6.Cg-Tg(Prrx1-cre)1Cjt/J (Prrx1-Cre, JAX stock #005584, The Jackson Laboratory, Bar Harbor, ME, USA),^33^ B6.Cg-Tg(Prrx1-cre/ERT2,-EGFP)1Smkm/J (Prrx1CreER^T2^, JAX stock #029211),^71^ B6.Cg-*Acan*^*tm1(Cre/ERT2)Crm*^/J (Acan-CreER^T2^, JAX stock #019148),^72^ B6.Cg-Gt(ROSA)26Sor^tm6(CAG-ZsGreen1)Hze^/J (Ai6, JAX stock #007906),^73^ and B6.TgROSA26^Bmp2-IRES-eGFP^ (Tg-Bmp2)^74^ have been previously described.

Apex1^tm1a/+^ and Apex1^tm2c/+^ were obtained at Transgénesis (Centro Nacional de Biotecnología, CNB) using a EUCOMM/KOMP-CSD knockout first allele strategy.^32^ E2.5 embryos were collected form superovulated CD1 female mice. Each embryo was microinjected with 4-6 targeted ES cells. Groups of 14-17 microinjected embryos were transferred into pseudopregnant CD-1 females. Twenty high-contribution (>95%) chimeras from two clones were identified based on coat pigmentation and two of them were confirmed positives after genotyping and backcrosses with C57Bl/6 J females to obtain the tm1a knockout - first allele.^75^ Apex1^tm1a/+^ mice were crossed with B6.Cg-Tg(ACTFLPe)9205Dym/J mice (JAX stock #005703)^76^ to obtain the Apex1^tm2c/+^ conditional allele.

*Apex1* reporter (Apex1^tm1a^) and conditional knockout mice (Apex1^tm2c^, denoted as Apex1^C/+^) were received as heterozygous. To obtain homozygous conditional mice, Apex1^C/+^ males and females were mated.

To silence Apex1 in limb mesenchyme, Apex1^C/C^ females were mated with heterozygous Prrx1-Cre males. Prrx1-Cre^+/-^;Apex1^C/+^ males were then crossed with Apex1^C/C^ females, to obtain Prrx1-Cre^+^;Apex1^C/C^ mice (denoted as P-Apex1^KO^). Wild type (WT) littermates were used as control group for subsequent experiments (Prrx1-Cre^+/-^;Apex1^C/+^, Apex1^C/C^, and Apex1^C/+^).

With the objective of generating a reporter mouse for Cre recombinase activity over chondrogenic lineage, homozygous, inducible Acan-CreER^T2^ mice were mated with Rosa26^Zsgreen^. All the F1 were Acan-CreER^T2+/-^;Rosa26^Zsgreen^ mice (denotated as A-ROSA^Zsgreen^).

To silence *Apex1* in the chondrogenic lineage during fracture healing, Apex1^C/C^ females were crossed with homozygous Acan-CreER^T2^ males. Acan-CreER^T2+^;Apex1^C/+^ males were mated with Apex1^C/C^ females, obtaining a 25% of Acan-CreER^T2+/-^;Apex1^C/C^ mice (denotated as A^ER^-Apex1^KO^). WT littermates were used as control group for subsequent experiments (Acan-CreER^T2+/-^;Apex1^C/+^, Apex1^C/C^, Apex1^C/+^).

Animals were housed in a barrier facility with a 24-h light/dark cycle and fed with standard mice chow. Mice were given *ad libitum* access to food and water.

### Non-stabilized closed tibial fracture model

All procedures were performed under general anesthesia, isoflurane (2%) (B. Braun Vet Care, Tuttlingen, Germany). Non-stabilized closed tibial fractures were performed in 7-12-week-old mice using a three-point bending device with a standardized force.^17^ Fractures were performed in the diaphyseal segment of left tibiae, and confirmed radiologically. Post-operative pain was controlled using subcutaneous injections of buprenorphine (0.05 mg/kg body weight). For studying appendicular skeletal development, 4-week-old and 9-week-old animals were sacrificed. For studying fracture healing process, adult mice were sacrificed at 1-, 3-, 6-, 10-, 14, or 21-days post fracture (-dpf) by carbon dioxide (CO_2_).

### Unicortical bone defect

For unicortical bone defects, after anesthesia, the skin of the lateral side of the femur was shaven and disinfected, a longitudinal incision 2 cm long was made through the skin. Muscle fascia was opened, inserting muscles were gently mobilized along the lateral femoral shaft, taking care to leave the periosteum intact except for the drilling area. A unicortical subcritical defect was created using a 0.8 mm drill. Drilling was performed at low rates of rotation with the minimum pressure required, and the area was constantly irrigated with cool saline to prevent thermal injury. Inserting muscles were reapproximated, the skin closed, and mice were allowed to recover. Animals were sacrificed at 9-, 11- 14- and 25-days post-surgery (-dps) by carbon dioxide.

For rescue experiments, after drilling an absorbable collagen sponge containing 100 ng of rhBMP2 (InductOs, Medtronic, Dublin, Ireland) was implanted in the defect.

### Micro computed tomography (μCT) analysis

To perform the μCT analysis, 3D tomographic images of mouse tibiae were acquired using X-ray micro-CT (Quantum-GX, Perkin Elmer) with the following parameters: 90 kVp X-ray source voltage, 88 μA current and high-resolution scan protocol for a total acquisition time of 14 min and a gantry rotation of 360 degrees. The plugins integrated in BoneJ were applied to the trabecular segmentation mask obtained (Supplemental methods). Bone density was calculated using a methacrylate phantom which includes four cylinders with different concentrations of hydroxyapatite (0, 50, 250 and 750 mg HA/cc).

To perform the callus analysis, a ROI containing the whole callus (10 × 10 x 10 mm) was reconstructed from the original scan at a resolution of 20 microns per voxel using the Quantum 3.0 software. Histomorphometric analysis in each ROI was carried out using a plugin developed for Fiji/ImageJ. The mentioned plugin was developed by the Imaging Platform at the Center for Applied Medical Research (CIMA). For fracture callus morphometric analysis, CT images were automatically reconstructed using the Cobra software (Exxim Computing Corporation, Pleasanton, CA). 3D bone images were rendered using the Amira 3D Software for preclinical analysis (Thermo Fisher Scientific, Waltham, MA, USA).

### Tamoxifen treatment

Tamoxifen (Sigma T5648) was mixed with absolute ethanol until completely dissolved. The Tamoxifen ethanol solution was added into a proper volume of corn oil (Sigma C8267) to a final concentration of 20 mg/mL. The ethanol was evaporated completely from the tamoxifen-ethanol-corn oil mixture using a speedVac centrifuge.

For tamoxifen treatment, animals received a close fracture of the tibia and tamoxifen was administered intraperitoneally. For AcanCreER^T2^, Tamoxifen (0.1 mL) was injected at 2-, 4- and 6-dpf (days post fracture); for Prrx1CreER^T2^, Tamoxifen (0.1 mL) was injected for three consecutive days after weaning or at 0-, 1- or 2-dpf.

### Histological processing of the samples

For appendicular skeleton development and fracture repair process analysis, mice were sacrificed, right tibiae and fractured left tibiae were dissected at the indicated times, washed with PBS, and fixed for 24 h at RT in a 4% PFA solution. After fixation, tibiae were decalcified, dehydrated through graded alcohol series and embedded in paraffin. Embedded tibia as well as fracture calluses were serially sectioned at 4 μm thickness employing a microtome (HM 340 E, Micron). Histological evaluation of non-fractured hind limbs and fracture calluses was performed with Hematoxylin & Eosin (H&E) and Safranin O/Fast green (SO) staining on selected sections (1 of every 10 slides), respectively.^15^

### Immunohistochemistry and immunofluorescence

For Ki-67 immunohistochemical assay (1:100, Leica Biosystems, # NCL-L-Ki67, Nussloch, Germany), sections were treated for antigen unmasking 30 min at 95 °C with EDTA antigen retrieval buffer (10 mmol/L Tris base, 1 mmol/L EDTA, pH 9) in a pressure cooker. Staining was developed using peroxidase with DAB substrate (EnVision, Dako, Glostrup, Denmark).

Double immunofluorescence for type II collagen (1: 2 000, MP Biomedicals, #631711)/ type X collagen (1:100, Millipore, #234196), type II collagen/ MMP-9 (1:100, Millipore, # ABT544) or MMP-13 (1:100, Invitrogen, # MA-14238)/type X collagen was performed via sequential antigen unmasking with 4 mg/mL of hyaluronidase (Sigma-Aldrich, St. Louis, MO, USA) in PBS, pH 5.5 for 15 min at 37 °C, and 4 mg/mL of pepsin (Sigma-Aldrich) in HCl 0.01 mol/L for 30 min at 37 °C. Immunofluorescence for Endomucin (1:50, Santa Cruz, #SC-65495) and double immunofluorescence for Endomucin/MMP-9 were performed via antigen unmasking with 1 mg/mL of trypsin and 1 mg/mL of CaCl_2_ in PBS for 1 h at 37 °C. In all cases, sections were incubated overnight with primary antibodies at 4 °C in a humidified chamber. Primary antibody was washed with PBS and slides were incubated with Alexa Fluor®488 (Invitrogen, #A11029/A11034), Alexa Fluor®568 (Invitrogen, #A11004/A11011), or AlexaFluor®594 (Invitrogen, #A11007) secondary antibody for 1 h at RT. All preparations were counterstained employing a solution 1:1 of DAPI-Glycerol (Thermo Fisher Scientific). Preparations were mounted with VECTASHIELD^®^ Antifade Mounting Medium (Vector Laboratories, Newark, CA).

### TRAP staining

For the TRAP analysis, the Leukocyte Acid Phosphatase (TRAP) Kit (387A-1KT, Sigma-Aldrich) was employed. TRAP staining was performed following manufacturer instructions.

### Histomorphometric analysis

Bright-field digital images of histological preparations were acquired employing an Aperio CS2 scan (Leica Biosystems, Nussloch, Germany). Immunofluorescent images were acquired with a Vectra Polaris multispectral image acquisition system (Perkin Elmer, Waltham, MA, USA). Histological preparations, TRAP staining, and immunofluorescence preparations were quantified using ImageJ/Fiji software, employing custom macros. The signal was quantified as total area (arbitrary units) or distance (μm). Illustrations were created using BioRender© (https://app.biorender.com). Illustrations and digital images were imported into Adobe Photoshop and formatted. Figures were created employing Adobe Photoshop.

### RNA extraction

For RNA extraction, calluses were ground using liquid nitrogen and a pestle. Total RNA from ground calluses was extracted employing TRIzol (Life Technologies, Carlsbad, CA, USA) following manufacturer instructions. After chloroform extraction, total RNA was purified from the aqueous solution using RNeasy Plus Mini Kit (Qiagen, Hilden, Germany) following manufacturer instructions.

### Bulk RNA-seq

RNA quality was measured employing the kit “High Sensitivity RNA ScreenTape” and following manufacturer instruction. All RNA samples presented a high RNA quality (RNA integrity numbers between 7 and 9) and were adequate for further transcriptomic analysis. Roughly 150 ng of high-quality total RNA were used for transcriptomic interrogation of WT and P-Apex1^KO^ fracture calluses using Illumina’s Stranded Total RNA Prep Ligation with Ribo-Zero Plus (Illumina, San Diego, CA, USA) according to the manufacturer’s instructions. Sequencing was carried out in an Illumina NextSeq 2000 (Illumina) at a depth of 50 million reads per sample. Samples were demultiplexed using and aligned to the mouse genome (mm39) with STAR (V2.7.0 d). Gene expression quantification was performed using the featureCounts function implemented in the R package. The filterByExpr function implemented in the edgeR package was used to filter out genes with low number of counts for downstream analyses. Differential expression analyses were performed with DESeq2 (v1.32.0). Gene Set Enrichment analysis (GSEA) was performed with fgsea (v.1.18.0). For pathway enrichment, we considered the Hallmark, KEGG and Gene Ontology biological processes databases. The gene sets were obtained using msigdbr (v.7.4.1). The resulting p-values were corrected with Benjamini-Hochberg, setting a 0.05 cutoff. Detailed procedures are listed in the Supplemental Methods.

### Quantitative RT-PCR (qPCR)

For qPCR, 0.5 to 1 μg of total RNA was retrotranscribed using qScriptTM Supermix (Quantabio, Gaithersburg, MD). The qPCR was performed in a QuantStudio 5 PCR system (Applied Biosystems, Foster City, CA, USA) using Taqman actin beta probe (Mm02619580_g1, Life Technologies) as reference gene. Relative expression of genes of interest was calculated using the ΔΔCt method.

The Taqman probes used (Life Technologies) are listed in Supplemental Methods, Table S3.

### Statistical analysis

Graphical results are expressed as median and interquartile range in box and whiskers representing maximum and minimum values. Statistical analysis was performed using GraphPad Prism 10.0 software (GraphPad Software Inc., La Jolla, CA, USA). Data distributions were analyzed employing Kolmogorov-Smirnov test. Single comparisons were analyzed by Student t-test or Mann Whitney U test. For multiple comparisons, we employed one-way ANOVA or Kruskal Wallis test followed by Sidàk’s or Dunn’s post hoc analysis, respectively. Significance was set at *P* < 0.05.

## Supplementary information

Supplementary Figures

Supplemental methods

Supplemental Table 1

Supplemental Table 2

## Data Availability

The RNA-seq data generated in this study have been deposited in the Gene Expression Omnibus (GEO) under accession number GSE278433 (token for anonymous access by the reviewers: uvwlqmykzlgfneh). The authors declare that the rest of the data supporting the findings of this study are available after reasonable request to the corresponding author.
