# Supplementary material for: APEX1, a transcriptional hub for endochondral ossification and fracture repair
Source: Bone Res. 2026 Jan 16;14:7. doi: 10.1038/s41413-025-00486-1 (PMC12811256; doi:10.1038/s41413-025-00486-1)
Supplement: Supplementary file 1 — Supplementary Figures [file 41413_2025_486_MOESM1_ESM.pdf]

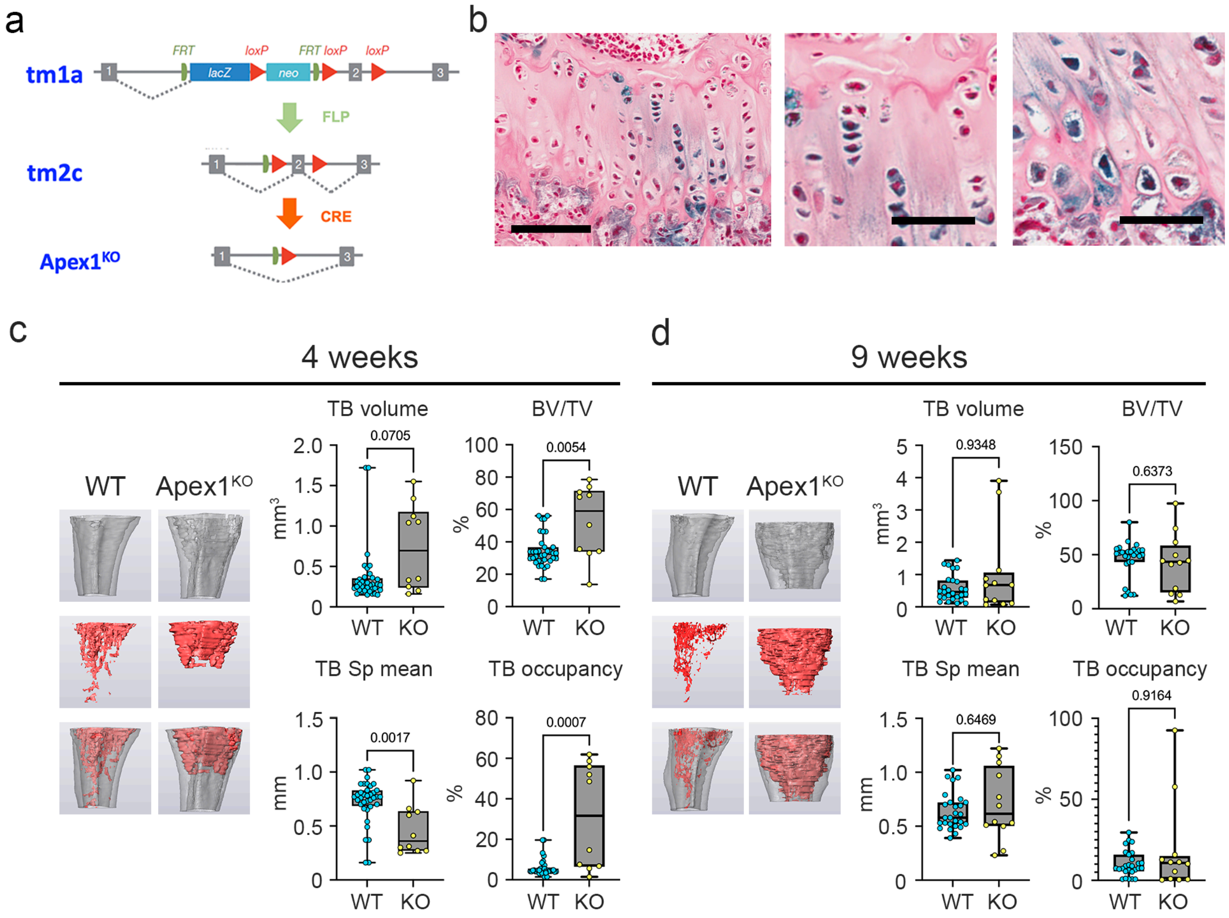

Supplemental figure 1. (**a**), *Apex1* first allele knockout strategy (**b**), Expression dynamics of *Apex1* in the appendicular skeleton. Representative β-gal staining of 9 weeks-old Apex1^{tm1c} mice tibia showing positive staining in the growth plate. Left panel, general view of the growth plate shows positive staining of chondrocytes at different differentiation stages. Scale bar, 1 mm. Center panel, magnification of resting and columnar chondrocytes positive for β-gal staining; right panel, hypertrophic chondrocytes. Scale bar, 100 µm. (**c**), Morphometric analysis of the metaphysis for 4 weeks-old mice tibias (WT, n = 36; P-Apex1^{KO}, n = 10). (**d**), Morphometric analysis of the metaphysis for 9 weeks-old mice tibias (WT, n = 27; P-Apex1^{KO}, n = 12). Length, tibia length; volume, total bone volume; density, average bone density. TB, trabecular bone; BV/TV, bone volume/total volume. Data are represented as median and interquartile range; whiskers represent maximal and minimal values. P values were determined by two-tailed Student's t test.

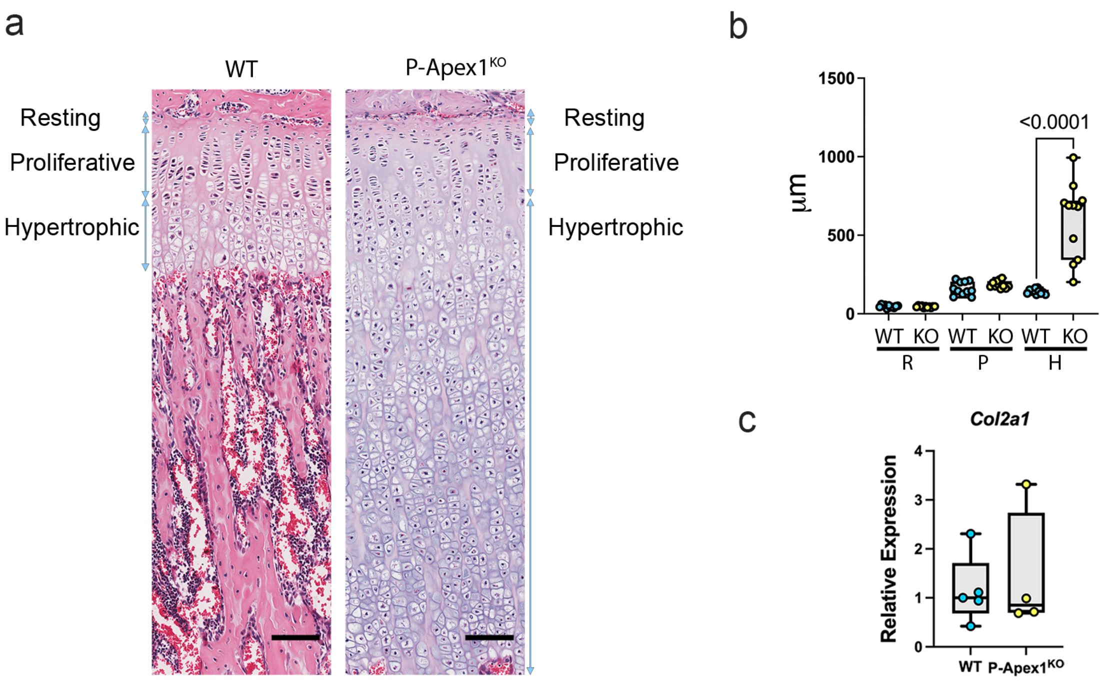

Supplemenental figure 2. (**a**), H&E staining showing differences in the length of the growth plate of 4 week old P-Apex1^{KO} and WT littermate mice. Size bar, 100 µm. (**b**), Zone length quantification confirmed that significative differences were localized in the hypertrophic zone. P value were determined by one way ANOVA (WT, n = 11; P-Apex1^{KO}, n = 11; $p < 0.0001$) followed by Sidàk's multiple comparisons test. (**c**), The expression levels of *Col2a1* in 4-week-old metaphysis was determined by qRT-PCR showing no significative differences between P-Apex1^{KO} and WT littermates.P value was determined by Mann Whitney test (WT, n = 5; P-Apex1^{KO}, n = 4; p = 0.9048).

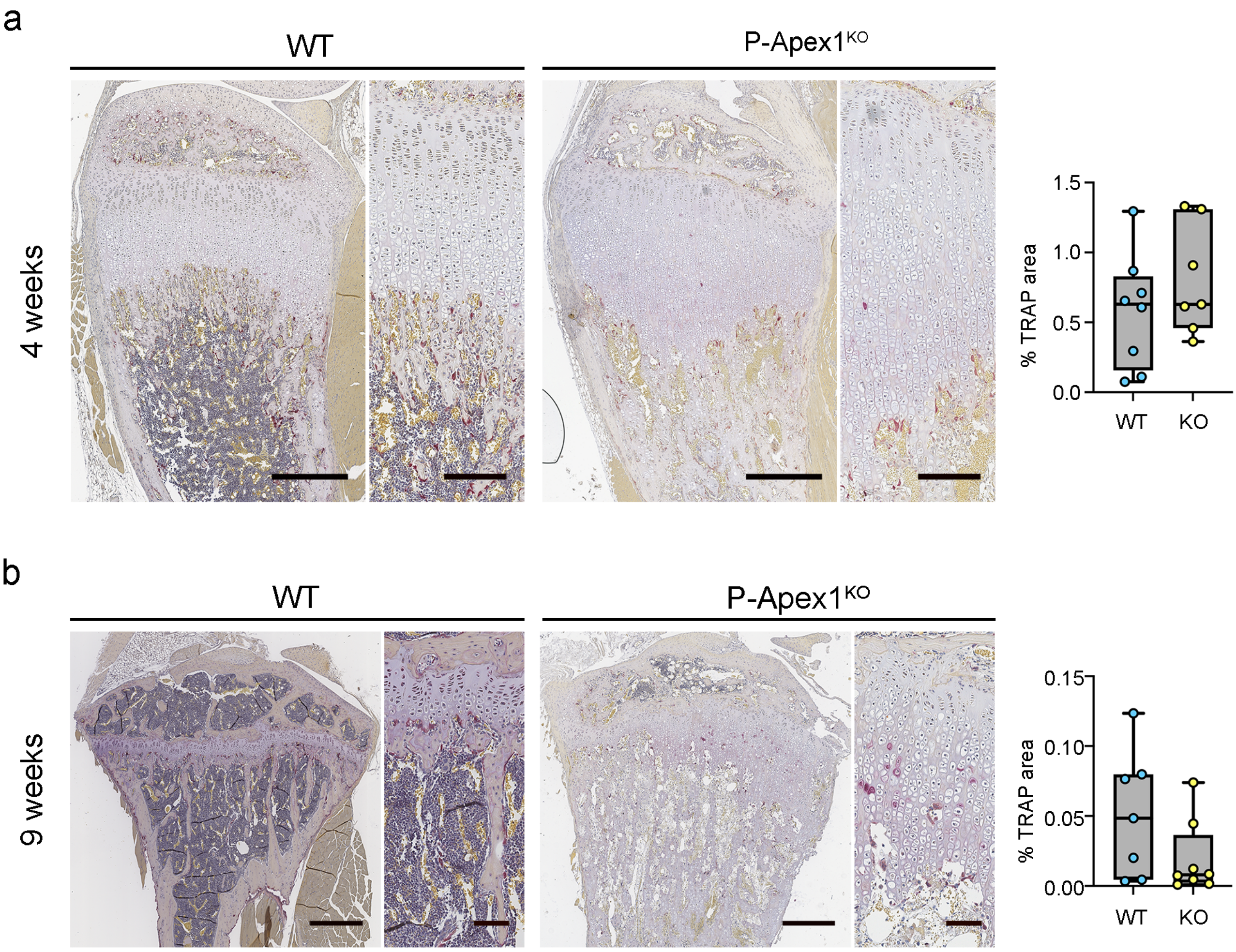

Supplemental figure 3. ***Apex1 silencing does not impair osteoclasts/chondroclasts recruitment to the growth plate.*** Representative images of sagittal sections of the proximal tibia and quantification of TRAP-stained areas at 4-weeks old (**a**) or 9-weeks old (**b**) WT and P-Apex1^{KO} animals. Left panels, whole view of the proximal tibia; scale bar, 1 mm. Right panels, magnification view of the growth plate and subjacent trabecular bone; scale bar, 100 µm. Quantification of the TRAP positive areas showed no significative differences between groups in 4 weeks-old animals (WT, n = 8; P-Apex1^{KO}, n = 7); p = 0.2993 by Student's t-test, or 9-weeks-old animals (WT, n = 7; P-Apex1^{KO}, n = 8); p = 0.1520 by Mann-Whitney test. Results are presented as median with interquartile range; whiskers represent maximum and minimum values.

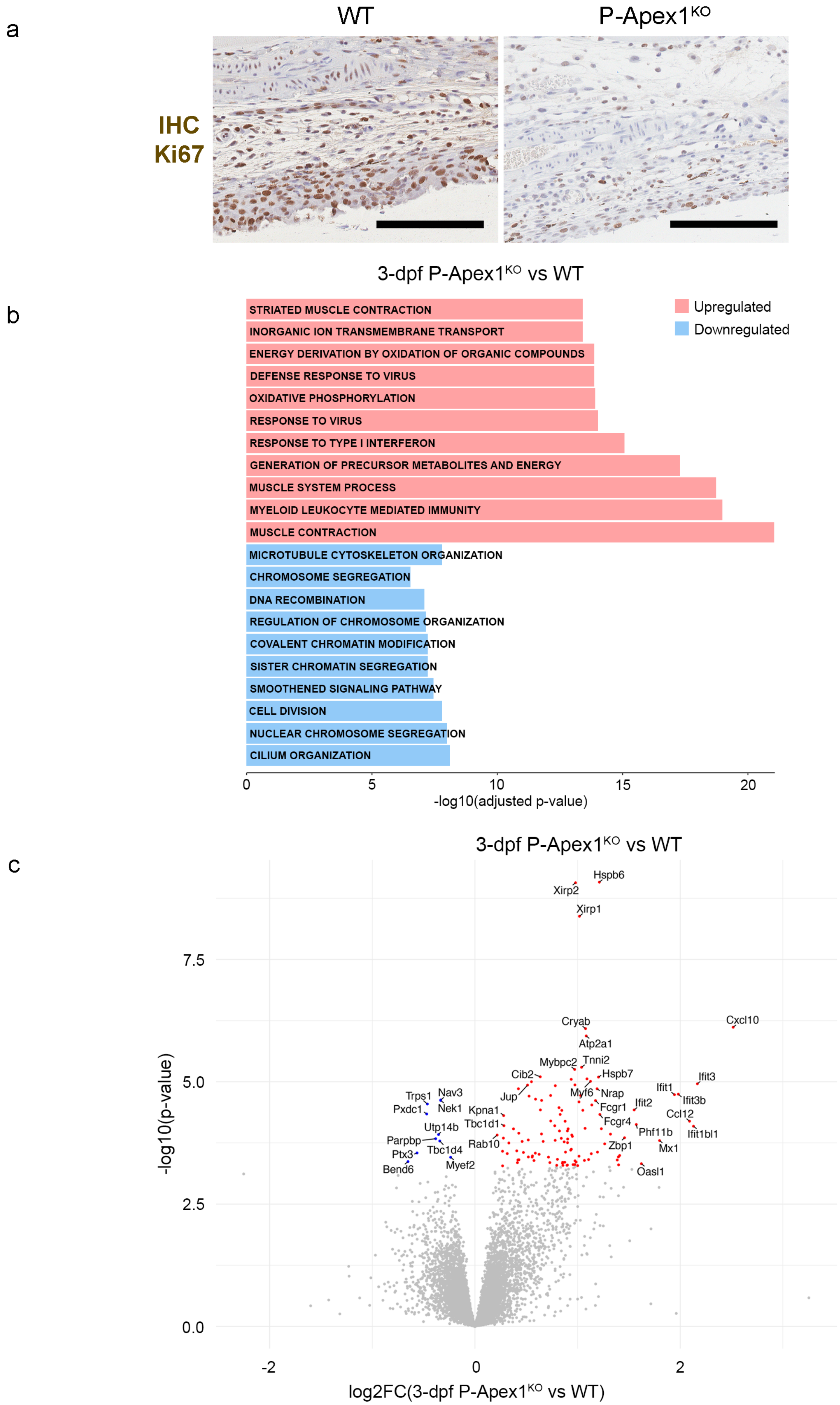

Supplemental figure 4

Supplemental figure 4. ***Transcriptomic analysis of 3-dpf fracture calluses.*** (**a**), Representative images of the immunohistochemistry against Ki67 in P-Apex1^{KO} and WT littermate mice. Scale bar, 150 µm. (**b**), Gene ontology analysis (Biological Process database) showing top 10 significantly altered key biological processes between P-Apex1^{KO} (n = 4) and WT littermates (n = 4) . (**c**),Volcano plot of the transcriptome wide analysis from P-Apex1^{KO} (n = 4) and WT littermates (n = 4).

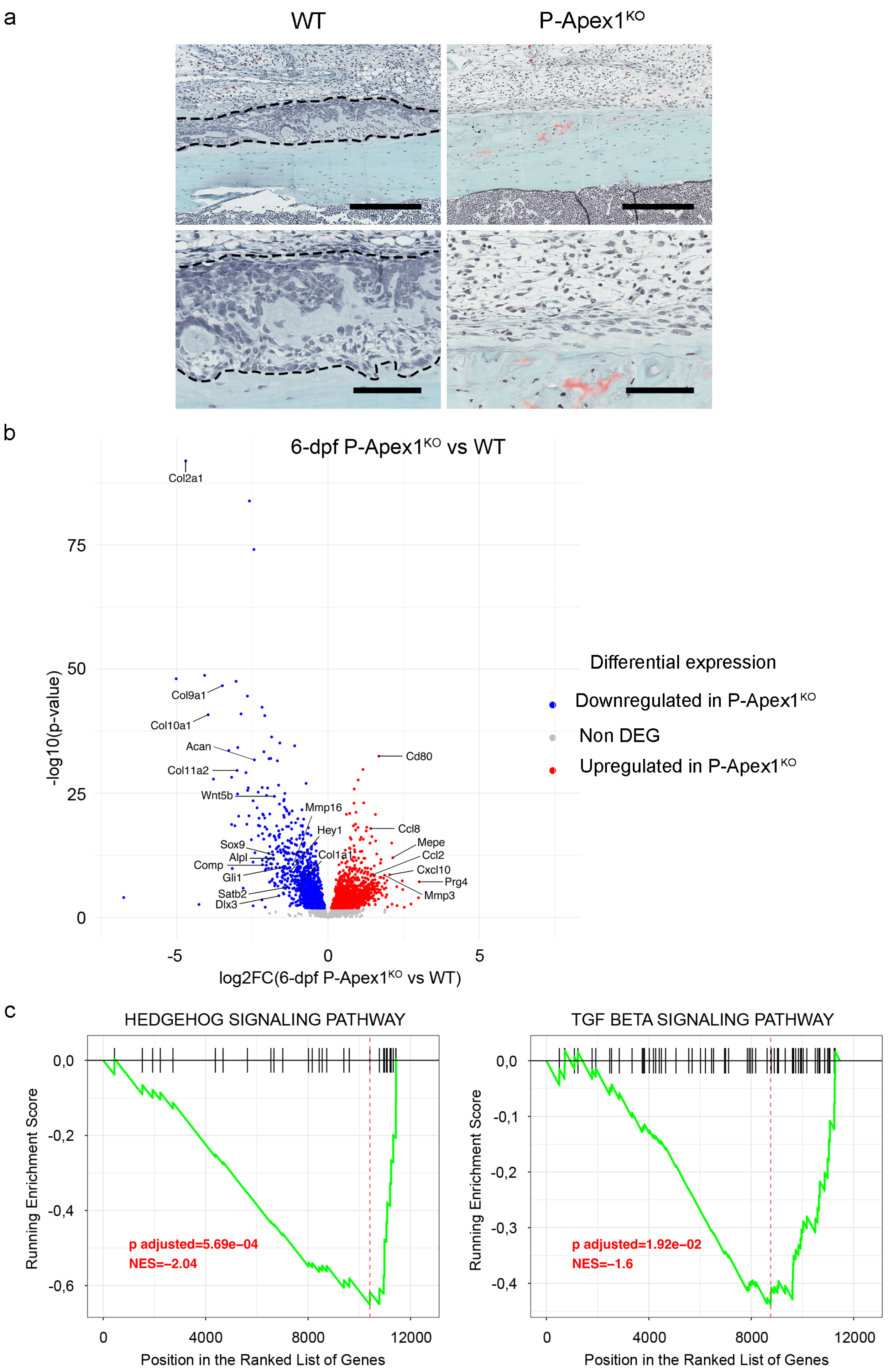

Supplemental figure 5

Supplemental figure 5. ***Impact of Apex1 silencing in the early reparative phase.*** (**a**), Reduced intramembranous bone formation in P-Apex1^{KO} mice during the early reparative phase, 6-dpf, as visualized by histological analysis. Dotted black line limits the cancellous bone. Scale bar, upper panels 300 µm; lower panels, 100 µm. (**b**), Volcano plot for the transcriptome wide analysis from P-Apex1^{KO} (n = 3) and WT littermates (n = 5) derived calluses at 6-dpf. Crucial chondrogenic markers are labeled. (**c**), Gene set enrichment analysis showing the impact of Apex1 silencing during the early reparative phase over Hedgehog- and TGF-β/BMP-signaling pathways.

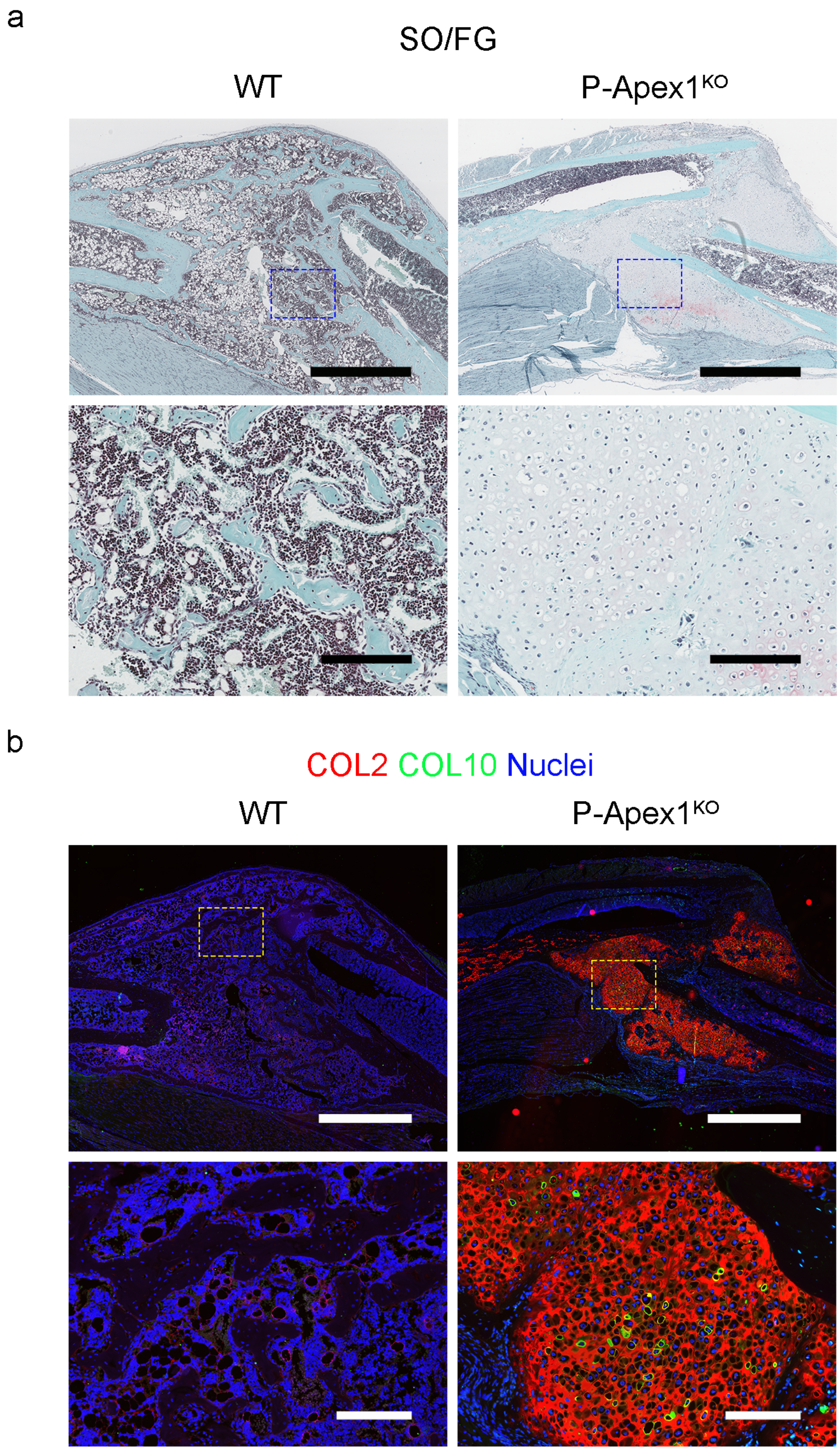

Supplemental figure 6. ***Impaired cartilage resorption at 21-dpf in P-Apex1^{KO} mice.*** (**a**), Safranin O/Fast Green (SO/FG) staining in P-Apex1^{KO} mice 21-dpf fracture calluses showed the presence of cartilaginous tissue with reduced content of Safranin O. Scale bar, upper panels, 1.5 mm; lower panels, 200 µm. (**b**), The chondrogenic nature of the calluses in P-Apex1^{KO} mice was confirmed by immunohistological analysis against the presence of type II and type X collagen. Scale bar, upper panels, 1.5 mm; lower panels, 200 µm.

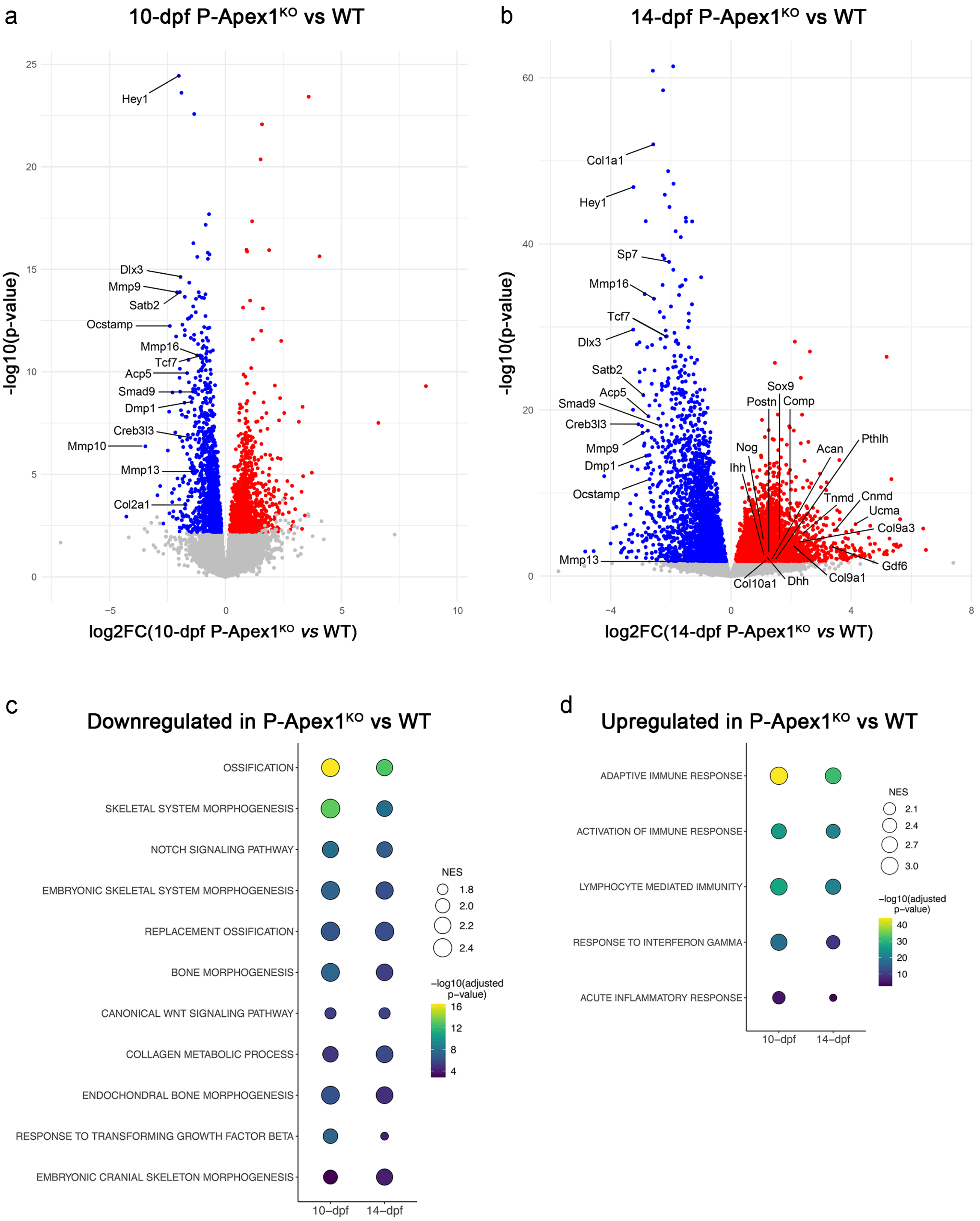

Supplemental figure 7

Supplemental figure 7. ***Transcriptomic analysis of the late reparative phase.*** Volcano plots and gene set enrichment analysis (GSEA) considering the Gene Ontology Biological Processes database showing an altered endochondral ossification progression at 10- (**a**) and 14-dpf (**b**). (**c**), Downregulated processes in P-Apex1^{KO} vs WT animals at 10- and 14-dpf. (d), Upregulated processes in P-Apex1^{KO} vs WT animals at 10- and 14-dpf.

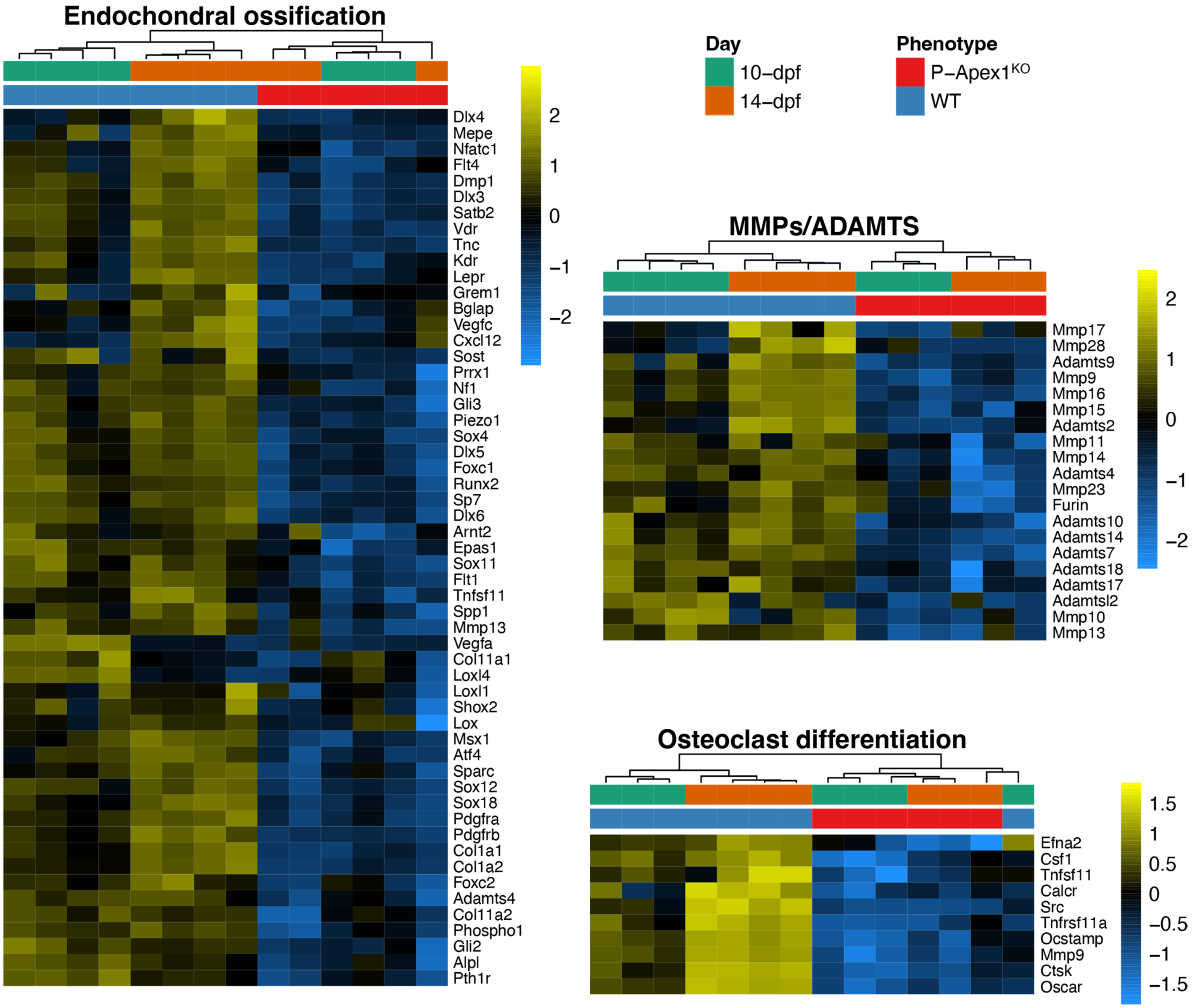

Supplemental figure 8. ***Impaired progression of endochondral ossification in P-Apex1^{KO} mice.*** Heatmap including gene names for the DEG (Figure 5b) related with endochondral ossification and replacement ossification (genes related with MMPs/ADMATSs and osteoclasts differentiation).

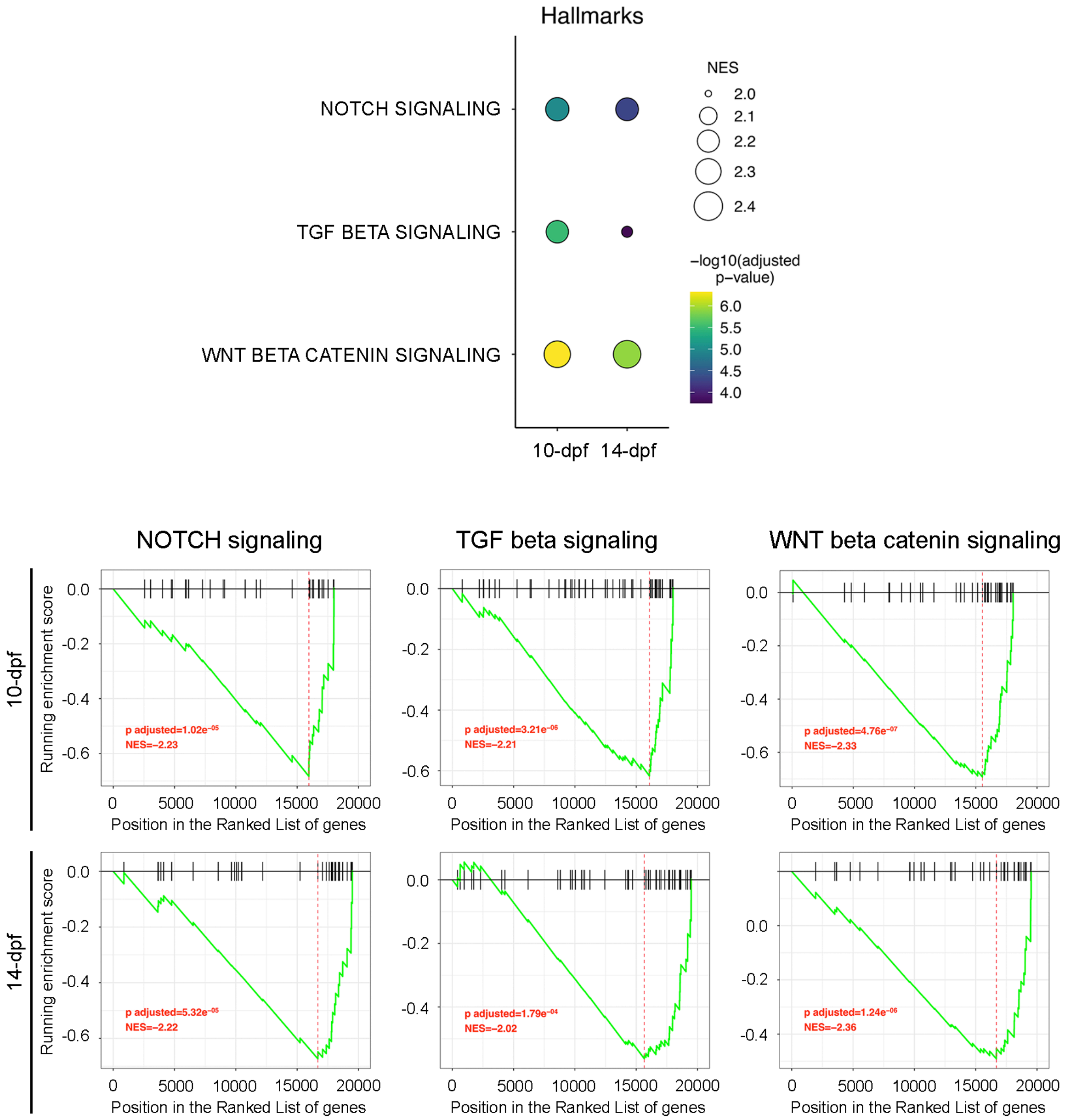

Supplemental figure 9. ***Transcriptomic analysis for key signaling pathways during endochondral ossification***. GSEA showed that major signaling pathways NOTCH, BMP and WNT were downregulated in P-Apex1^{KO} mice at the end of the reparative phase.

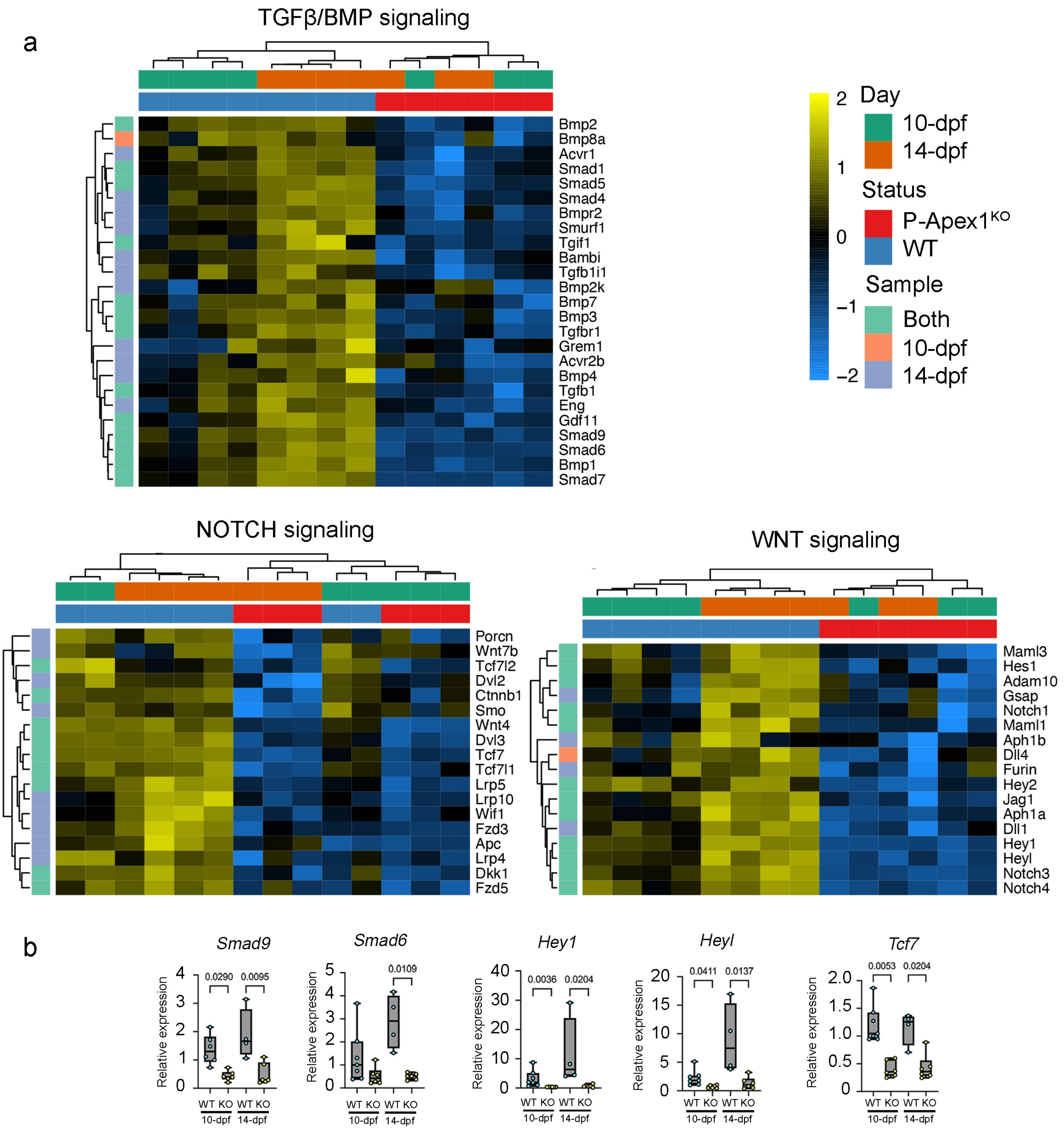

Supplemental figure 10. (**a**), *Full histogram containing DEG for key signaling pathways during endochondral ossification* (Figure 5d). (**b**), Quantitative RT-PCR analysis validating the bulk RNA-seq data, all results are expressed as median and interquartile range; whiskers represent maximum and minimum values. 10-dpf (WT, n = 7; P-Apex1^{KO}, n = 6), 14-dpf (WT, n = 4-7; P-Apex1^{KO}, n = 6). P values were determined by Kruskal Wallis test (*Smad9*, p = 0.0030; *Smad6*, p = 0.0086; *Hey1*, p = 0.0006; *Heyl*, p = 0.0028; *Tcf7*, p = 0.0011) followed by Dunn’s multiple comparisons test.

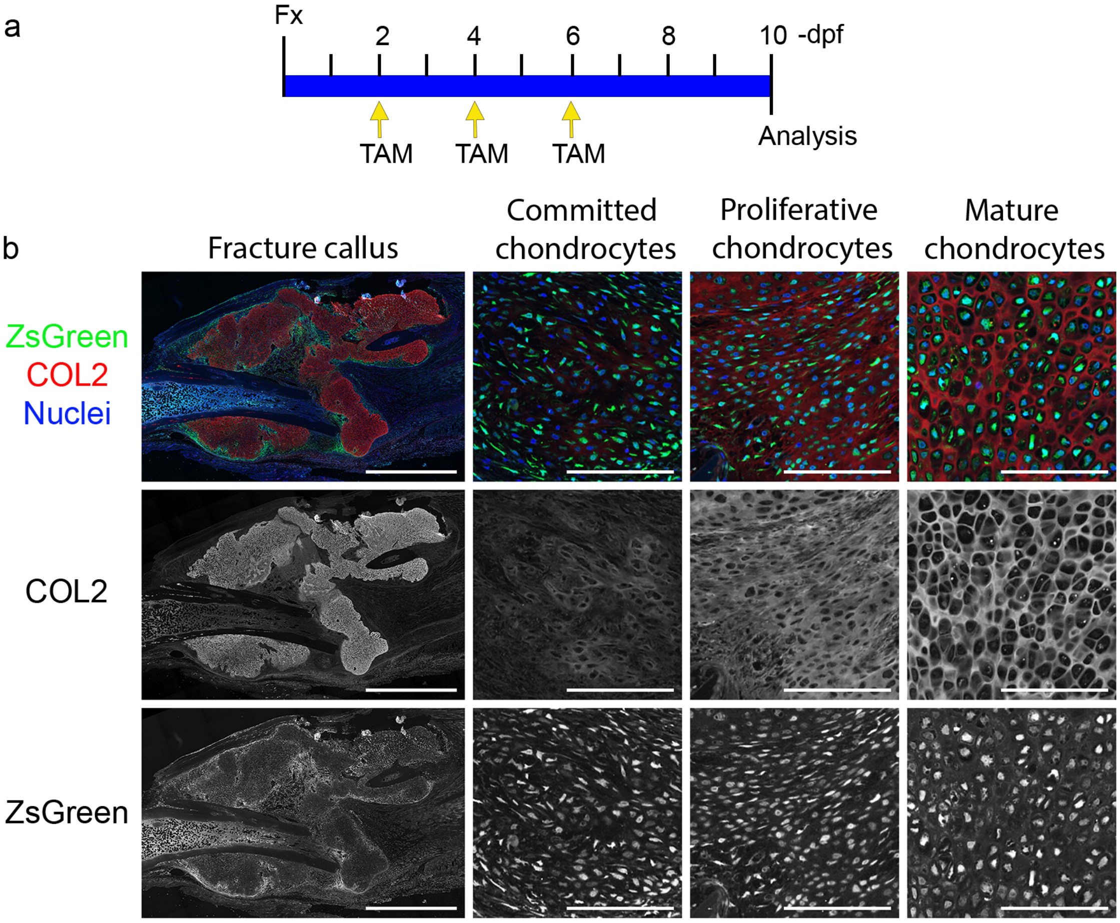

Supplemental figure 11. ***Strategy for Apex1 specific silencing in chondrocytes***. (**a**), Experimental design for CreER^{T2} induction. (**b**), Representative images of 10-dpf callus showing the recombination driven by Cre in chondrocytes at different stages of differentiation. Left panel, scale bar 1 mm. Right panels, scale bar 100 µm. COL2, type II collagen.

The aggrecan promoter drives Cre mediated recombination in committed chondrocytes and derived chondrocyte populations after tamoxifen induction. Because chondrocyte differentiation starts at the end of the inflammatory phase, we sought to define the mesenchymal populations affected by AcanCreER^{T2} recombination. We crossed AcanCreER^{T2} mice with ZsGreen reporter mice (ROSA^{ZsGreen}), doble hemizygous transgenic mice (AcanCreER^{2};ROSA^{ZsGreen}, A-ROSA^{ZsGreen}) received a close fracture of the tibia and tamoxifen at days 2-, 4- and 6-dpf. At 10-dpf animals were sacrificed and the presence of ZsGreen positive cells in the fracture callus were analyzed together to the immunohistological location of type II collagen. ZsGreen positive cells were detected across different cartilaginous locations as committed chondrocytes, delimiting type II collagen-stained areas, and as proliferative and mature chondrocytes embedded in type II collagen-stained areas. Scale bar, 1 mm (fracture callus, left column); right columns, 100 µm.

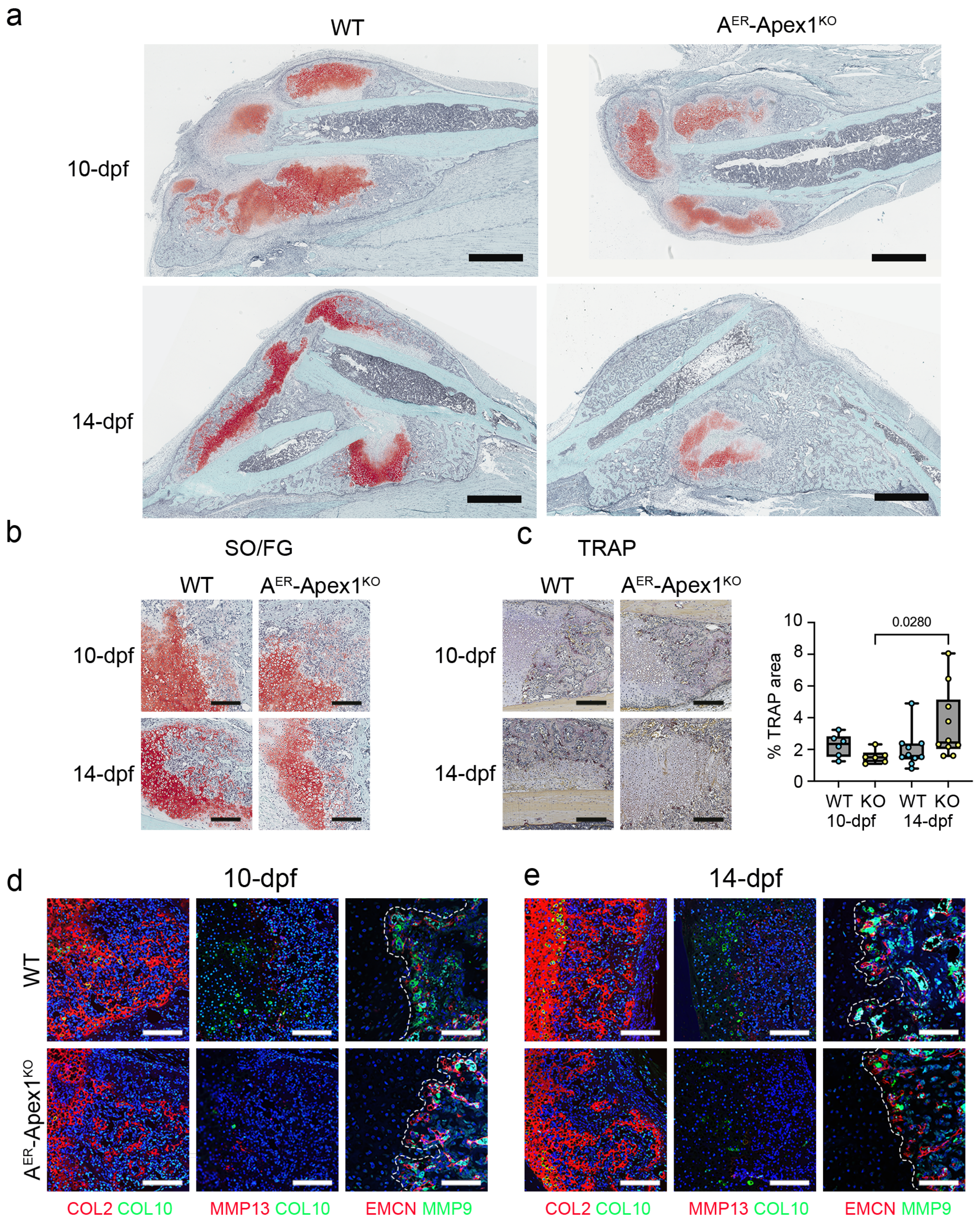

Supplemental figure 12. ***Histological and immunohistological analysis of A^{ER}-Apex1^{KO} fracture callus***. SO/FG staining showed reduced cartilage areas in A^{ER}-Apex1^{KO} calluses at 10- and 14-dpf (**a**) and signs of cartilage resorption in both A^{ER}-Apex1^{KO} and WT littermates at 10-and 14-dpf (**b**). (**c**), Silencing *Apex1* in chondrocytes has little impact in the recruitment and activity of osteoclasts. Results are expressed as median and interquartile range; whiskers represent maximum and minimum values. P values were determined by Kruskal-Wallis test ($p = 0.0296$) followed by Dunns multiple comparisons test. Immunohistological detection of markers of cartilage maturation and endochondral ossification progression for WT and A^{ER}-Apex1^{KO} at 10-dpf (**d**) and 14-dpf (**e**). Scale bar, 100 μm. COL2, type II collagen; COL10, type 10 collagen; EMCN, Endomucin. Dotted white lines label the chondro-osseous junction of the fracture callus.

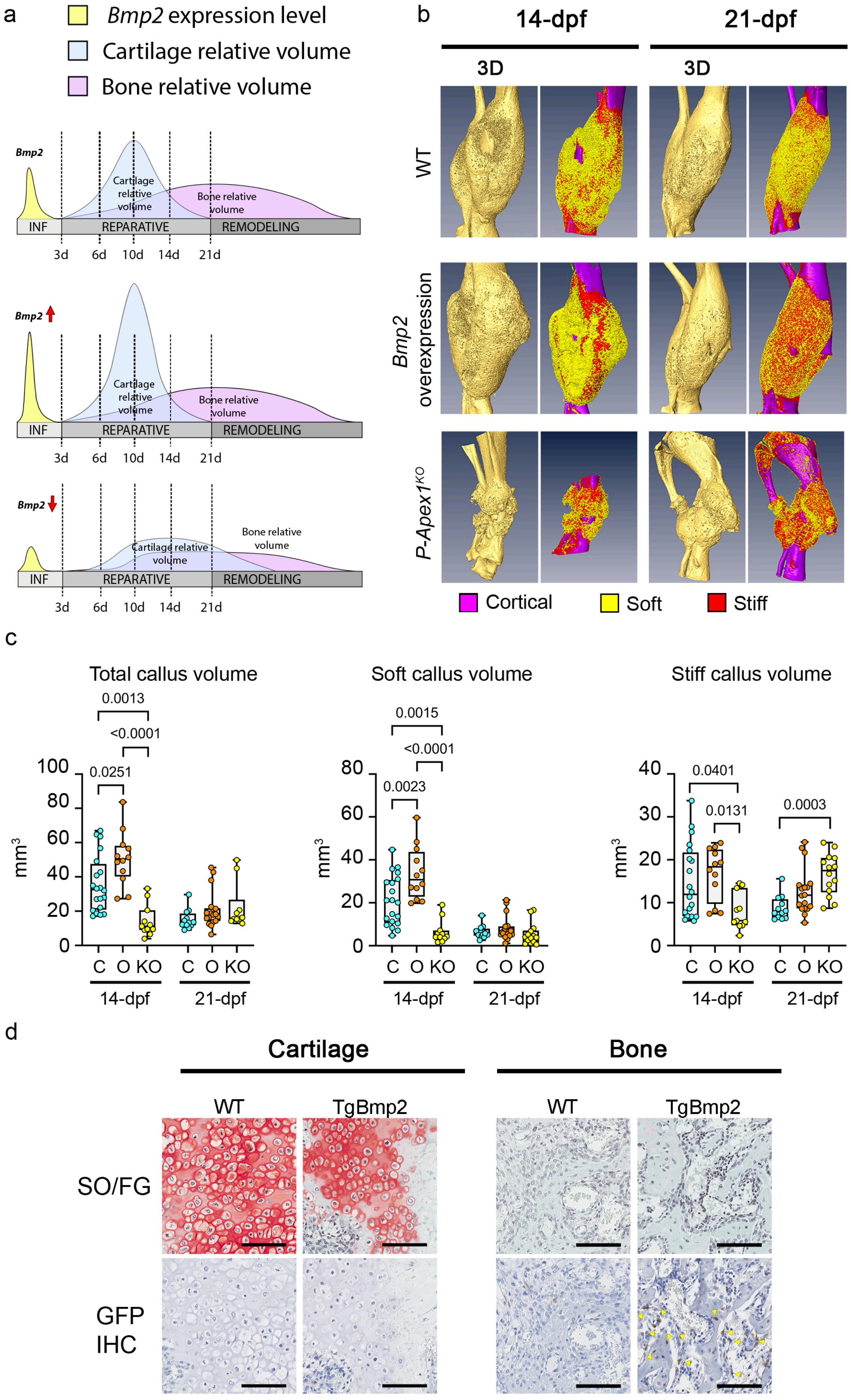

Supplemental figure 13

Supplemental figure 13. ***Bmp2 expression during the inflammatory phase impacts callus volume.*** (**a**), Graphical representation of the relation between levels of *Bmp2* expression, callus volumen, and *Apex1* silencing phenotype. (**b**), Three-dimensional reconstruction and segmentation of the fracture calluses at 14-dpf (WT (C), n = 20; *Bmp2* overexpression (O), n = 12; and P-Apex1^{KO}(KO), n = 11) and 21-dpf (WT (C), n = 12; *Bmp2* overexpression (O), n = 18; P-Apex1^{KO} (KO), n = 10). (**c**), Quantification of the total callus, soft (non-mineralized), and stiff (mineralized) callus volume. Results are expressed as median and interquartile range; whiskers represent maximum and minimum values. P values were determined by one way ANOVA (Total callus volume, $p < 0.0001$; Soft callus volume, $p < 0.0001$; Stiff callus volume, $p = 0.0493$) and Sidàk's multiple comparisons test. (**d**), Immunohistological validation of the strategy for *Bmp2* overexpression. The presence of an internal ribosomal entry site in the TgBmp2 mice allows GFP detection as reporter of *Bmp2* expression. The expression of GFP was assessed by inmmunohistochemistry at 14-dpf. Expression of *Bmp2* was detected in osteoblast/osteocytes along the callus (yellow arrowheads). Scale bar, 100 µm.
