# Supplementary material for: APEX1, a transcriptional hub for endochondral ossification and fracture repair
Source: Bone Res. 2026 Jan 16;14:7. doi: 10.1038/s41413-025-00486-1 (PMC12811256; doi:10.1038/s41413-025-00486-1)
Supplement: Supplementary file 2 — Supplemental methods [file 41413_2025_486_MOESM2_ESM.docx]

**Supplemental methods**

**X-gal staining**

The analysis of *Apex1* expression profile was performed using 9-weeks-old Apex1^tm1a/+^ mice.

To detect b-Galactosidase activity, whole mount Apex1^tm1a/+^ tibias were subjected to X-gal staining before histological processing and paraffin embedding as previously described^17^. After the incubation in X-gal solution, samples were fixed in 4% PFA and processed for histological analysis. Sections were counterstained with Nuclear Fast Red to allow histological evaluation and b-GAL profile analysis.

**Micro computed tomography (mCT) analysis**

For performing the μCT analysis, mice tibias 3D tomographic images were acquired using X-ray micro-CT (Quantum-GX, Perkin Elmer) with the following parameters: 90 kVp X-ray source voltage, 88 μA current and the high-resolution scan protocol for a total acquisition time of 14 minutes and a gantry rotation of 360 degrees. The plugins integrated in BoneJ were applied to the trabecular segmentation mask obtained. The tomographic three-dimensional images containing the whole bone had a total of 512 slices with isotropic 50 μm voxel size and a resolution of 512 pixels per slice. Histomorphometry analysis of the region of interest (ROI) containing the metaphysis (10 x 10 x 10 mm) was reconstructed from the original scan at a resolution of 20 microns per voxel using the Quantum 3.0 software. Trabecular histomorphometry parameters were calculated using BoneJ version 1.4.2.

**Bulk RNA-seq**

RNA quality was measured employing the kit “High Sensitivity RNA ScreenTape” and following manufacturer instruction. All RNA samples presented a high RNA quality (RNA integrity numbers between 7 and 9) and were adequate for further transcriptomic analysis. Roughly 150 ng of high-quality total RNA were used for transcriptomic interrogation of WT and P-Apex1^KO^ fracture calluses using Illumina’s Stranded Total RNA Prep Ligation with Ribo-Zero Plus (Illumina, San Diego, CA, USA) according to the manufacturer’s instructions. Briefly, cytoplasmic and mitochondrial rRNAs, as well as beta globin transcripts, were depleted from the samples. The remaining RNA was fragmented and reverse transcribed. To preserve strand specificity, a second strand cDNA synthesis step removed the RNA template while incorporating dUTP in place of dTTP. Next, double-stranded cDNA was A-tailed, then ligated to Illumina anchors bearing T overhangs. PCR-amplification of the library allowed the barcoding of the samples with 10-bp dual indexes and the completion of Illumina sequences for cluster generation. Libraries were quantified with Qubit dsDNA HS Assay Kit (Thermo Fisher Scientific) and their profile was examined using Agilent’s HS D1000 ScreenTape Assay (Agilent, Santa Clara, CA). Sequencing was carried out in an Illumina NextSeq 2000 (Illumina) using paired-end, dual-index sequencing (Read 1: 59 cycles; i7: 10 cycles; i5: 10 cycles Read 2:59 cycles) at a depth of 50 million reads per sample. Samples were demultiplexed using Illumina bcl2fastq software (v.2.2.0) and aligned to the mouse genome (mm39) with STAR (V2.7.0d). Gene expression quantification was performed using the featureCounts function implemented in the R package Rsubread (v2.4.3) counting uniquely mapped reads with reverse strandness. The Ensembl v104 gene annotation was considered as the reference. The filterbyExpr function implemented in the edgeR package was used to filter out genes with low number of counts for downstream analyses. Differential expression analyses with the Wald test and an FDR of 0.05 were performed with DESeq2 (v1.32.0) including the effect of sex in the analysis model. Gene Set Enrichment analysis (GSEA) was performed with fgsea (v.1.18.0), ranking genes by the Wald statistic. For pathway enrichment, we considered the Hallmark, KEGG and Gene Ontology biological processes databases. The gene sets were obtained using msigdbr (v.7.4.1). The resulting p-values were corrected with Benjamini-Hochberg, setting a 0.05 cutoff.

**Supplemental table 3**: Taqman probes used in the study.

| **Gene** | **Reference** |
| --- | --- |
| *Sox9* | Mm00448840_m1 |
| *Col2a1* | Mm01309565_m1 |
| *Col10a1* | Mm00487041_m1 |
| *Col9a1* | Mm00483836_m1 |
| *Col11a1* | Mm00483387_m1 |
| *Acan* | Mm00545794_m1 |
| *Col1a1* | Mm00801666_g1 |
| *Dmp1* | Mm01208363_m1 |
| *Dlx3* | Mm00438428_m1 |
| *Sp7* | Mm04933803_m1 |
| *Satb2* | Mm00507337_m1 |
| *Acp5* | Mm00475698_m1 |
| *Ocstamp* | Mm00512445_m1 |
| *Mmp16* | Mm00490659_m1 |
| *Mmp13* | Mm00439491_m1 |
| *Mmp9* | Mm00442991_m1 |
| *Mmp10* | Mm01168399_m1 |
| *Smad6* | Mm00484738_m1 |
| *Smad9* | Mm00649885_m1 |
| *Hey1* | Mm00468865_m1 |
| *Heyl* | Mm00516558_m1 |
| *Tcf7* | Mm00493445_m1 |
| *Bmp2* | Mm01340178_m1 |
